# Peripheral inflammatory response in people after acute ischaemic stroke and isolated spontaneous cervical artery dissection

**DOI:** 10.1038/s41598-024-62557-3

**Published:** 2024-05-27

**Authors:** Angelika Bauer, Christian Boehme, Lukas Mayer-Suess, Dagmar Rudzki, Michael Knoflach, Stefan Kiechl, Markus Reindl

**Affiliations:** 1 Clinical Department of Neurology, Medical University of Innsbruck, Innsbruck, Austria; 2 VASCage Research Centre on Vascular Ageing and Stroke, Innsbruck, Austria; 3 Institute of Hygiene and Medical Microbiology, Medical University of Innsbruck, Innsbruck, Austria

**Keywords:** Cytokine, Chemokine, Ischaemic stroke, Spontaneous cervical artery dissection, Outcome, Neurology, Stroke, Biomarkers, Chemokines, Cytokines

## Abstract

The systemic inflammatory response following acute ischaemic stroke remains incompletely understood. We characterised the circulating inflammatory profile in 173 acute ischaemic stroke patients by measuring 65 cytokines and chemokines in plasma. Participants were grouped based on their inflammatory response, determined by high-sensitivity C-reactive protein levels in the acute phase. We compared stroke patients’ profiles with 42 people experiencing spontaneous cervical artery dissection without stroke. Furthermore, variations in cytokine levels among stroke aetiologies were analysed. Follow-up samples were collected in a subgroup of ischaemic stroke patients at three and twelve months. Ischaemic stroke patients had elevated plasma levels of HGF and SDF-1α, and lower IL-4 levels, compared to spontaneous cervical artery dissection patients without stroke. Aetiology-subgroup analysis revealed reduced levels of nine cytokines/chemokines (HGF, SDF-1α, IL-2R, CD30, TNF-RII, IL-16, MIF, APRIL, SCF), and elevated levels of IL-4 and MIP-1β, in spontaneous cervical artery dissection (with or without ischaemic stroke as levels were comparable between both groups) compared to other aetiologies. The majority of cytokine/chemokine levels remained stable across the study period. Our research indicates that stroke due to large artery atherosclerosis, cardioembolism, and small vessel occlusion triggers a stronger inflammatory response than spontaneous cervical artery dissection.

## Introduction

Certain factors, such as the severity of the stroke and the patient's age which influence the high disability and mortality rates associated with stroke^1^, remain beyond our control^2^. However, other factors such as inflammation related to stroke, have been explored as new targets to prevent stroke-related complications and improve functional outcomes^3^. These concepts were adopted with the emergence of novel therapeutic targets aimed at reducing peripheral inflammation in individuals after myocardial infarction, since elevated cytokine levels have been linked to unfavourable clinical outcomes in ischaemic stroke patients^4^. Long-term studies have revealed systemic inflammation that emerges not only within minutes to hours after acute ischaemic stroke (AIS), but persists for several days and drives complications^5,6^. Although several receptor antagonists have been studied for their potential to reduce circulating inflammatory cytokine levels and improve clinical outcomes after AIS, their effectiveness remains uncertain. Large ongoing trials^7^ and conflicting results from smaller studies^8,9^, reflect our incomplete understanding of immune processes following cerebral ischaemia. Nevertheless, some ongoing trials show promise, such as the investigation of colchicine to prevent inflammation^10^.

C-reactive protein (CRP), a prominent acute-phase protein, is one of the most extensively studied blood markers for inflammation and is implemented in many laboratory panels used in people with AIS^11^. High CRP levels have been found not only to be associated with an increased risk of developing ischaemic stroke^12^, but also to predict functional outcome and mortality when measured at the time of admission^13–18^. Other prominent inflammatory markers in stroke research include interleukin (IL)-6, IL-1, IL-10, tumour necrosis factor (TNF)-α and TNF-β, as well as the chemokines monocyte chemoattractant protein (MCP)-1, macrophage inflammatory protein (MIP)-1α, and chemokine ligand-5^19–21^. Most of these cytokines and chemokines can be found upstream of CRP, but their effect on functional outcome is still not as sufficiently studied as the effect of CRP. Thus, more research is needed to clarify the role of these cytokines and chemokines in cerebrovascular disease, including their association with stroke severity, stroke aetiology, and functional outcomes. Also the precise impact of the post-ischaemic inflammatory response on brain recovery, especially in advanced stages, remains incompletely understood^6,22^.

In recent years, various smaller panels of pro- and anti-inflammatory cytokines and chemokines have been studied in patients with stroke. The aim of this study was to measure plasma levels of 65 different cytokines, chemokines, soluble surface molecules, and immune receptors using one methodology in patients with AIS to better understand the interplay between pro- and anti-inflammatory cytokines in the acute phase. Furthermore, we aimed to evaluate possible differences between patients showing a higher peripheral inflammatory response in the acute phase, as indicated by high CRP levels in the blood, and patients with normal CRP levels. We also investigated if levels of these 65 cytokines and chemokines remain stable in follow-up samples up to twelve months and examined their predictive value for functional outcome. Finally, we performed subtype-stratified analyses as only a few studies so far have assessed potential differences among various AIS aetiologies.

## Methods

### Samples, study population and routine laboratory diagnostics

The use of frozen plasma samples from a biobank for this study was approved by the local ethics committees of the Medical University Innsbruck, Austria (ReSect study: EK#UN5072, 325/4.1; STROKE-CARD study: EK#UN2013-0045). Written informed consent for diagnostic procedures and biological sample storage for research purposes was obtained from all patients or their legal representatives. All methods were performed in accordance with the relevant guidelines and regulations.

Samples from patients were included from these two separate cohort studies, the STROKE-CARD trial (post-stroke disease management trial investigating an unselected cohort of consecutive AIS patients treated at the Medical University of Innsbruck)^23,24^ and the ReSect study (cohort study of spontaneous cervical artery dissection (sCeAD) patients treated at the Medical University of Innsbruck)^25,26^. ReSect study participants were dichotomised as those suffering AIS due to sCeAD and those with local symptoms only (i.e. no ischaemic stroke; sCeAD-nonAIS). Immediately after the acute event during the baseline assessment, ischaemic stroke aetiology was assessed using the TOAST criteria^27^ and AIS patients were classified into stroke due to large artery atherosclerosis (LAA), cardioembolism (CE), small vessel occlusion (SVO) or sCeAD. Additionally, in the STROKE-CARD cohort, stroke aetiology was reassessed at the three months follow-up visit. Details of patient selection and recruitment strategies for both studies have already been published^23–26^, and details on exclusion and inclusion criteria for both studies (STROKE-CARD and ReSect) are listed in the Supplementary methods (Supplementary File 1). Blood samples in both studies were drawn after an overnight fast and at least 12 h of abstinence from smoking, typically at 8 am. The samples were then immediately used for routine testing. After centrifugation, plasma samples were stored in a biobank at − 80 °C within 3 h after blood sampling. Individuals with any clinical or laboratory symptoms of infection prior to blood sampling for this study were excluded. In addition, particularly for blood sampling at follow-up, individuals who reported having a current infection or a positive history of infection the weeks before were not included in the selected patient cohort analysed in the current study.

At − 80 °C stored plasma samples of 173 people after AIS and 42 samples of sCeAD-nonAIS were included. Patients after AIS were classified based on high-sensitivity CRP levels, an acute phase protein implemented in stroke diagnostics laboratory panels at the Medical University of Innsbruck, as either having CRP levels ≤ 5 mg/l or > 5 mg/l. This cut-off of 5 mg/l was determined according to the standard high-sensitivity CRP reference levels used in clinical routine diagnostics at the University Hospital of Innsbruck^28^. No other upstream inflammatory markers except for CRP levels were measured in Innsbruck during the emergency diagnostic work-up. The following baseline laboratory parameters were taken from the emergency blood panel determined at the time of admission, even before any acute procedure was performed: CRP, the international normalised ratio (INR) level, prothrombin-time (PT) activity, platelet count, D-dimer level, antithrombin-III (AT-III) activity, fasting glucose, and lipid levels. These laboratory parameters were also assessed at month three and month twelve follow-up visits, except for D-dimer and AT-III activity levels. Blood pressure levels above 140/90 mmHg, or above 130/85 mmHg in people with diabetes, renal impairment, or small-vessel disease, or those on antihypertensive medication, were defined as hypertension. Diabetes was defined by a HbA_1c_ ≥ 6.5%, fasting glucose levels ≥ 126 mg/dl, or non-fasting glucose levels ≥ 200 mg/dl. Dyslipidaemia was defined by low-density lipoprotein cholesterol (LDL-C) levels > 100 mg/dl or > 70 mg/dl in high-risk patients according to guidelines at the time of the studies.

To assess the stability of cytokines, chemokines, and related molecules, as well as their change during disease recovery, some people after AIS were clinically followed up for up to 12 months after the initial event, with plasma samples taken regularly. Specifically, plasma samples from 79 people after AIS were measured at both 3 and 12 months post-stroke. Among these 79 people, 17 belonged to the AIS group with CRP levels > 5 mg/l, and 62 belonged to the group with levels ≤ 5 mg/l. Unfortunately, only baseline samples were available for the remaining individuals.

### Classification of stroke severity and parameters of functional outcome after twelve months

For acute assessment of stroke severity, the National Institutes of Health Stroke Scale (NIHSS) was evaluated upon admission, along with the modified Rankin Scale (mRS) score. People were grouped based on their NIHSS scores into minor (0–5), moderate (6–15), or severe (≥ 16) stroke categories^29^.

Long-term functional outcomes based on the mRS were also assessed at the 12-month follow-up visit, during which plasma samples were taken. Missing mRS follow-up data in AIS patients without this 12-month follow-up visit was completed through retrospective chart review of all available electronic medical files. The collected mRS-follow-up data is addressed to as “mRS at last clinical follow-up”. Good functional outcome was defined as mRS ≤ 2.

### Measurement of plasma levels of 65 cytokines, chemokines, and related molecules

Multiplex bead-based immunoassays measuring 65 cytokines, chemokines, and related molecules, were performed according to the manufacturer’s instructions (Thermo Fisher Scientific, Waltham, MA, USA; Immune monitoring 65-plex human ProcartaPlex panel, cat. #EPX650-10065-901). In detail the following 65 analytes were measured: a proliferation-inducing ligand (APRIL), B cell activating factor (BAFF), B lymphocyte chemoattractant (BLC), TNF receptor superfamily member 8 (CD30), CD40 ligand (CD40L), neutrophil-activating protein 78 (ENA-78), Eotaxin, Eotaxin-2, Eotaxin-3, fibroblast growth factor (FGF-2), Fractalkine, granulocyte colony-stimulating factor (G-CSF), granulocyte–macrophage colony-stimulating factor (GM-CSF), growth related oncogene α (GROα), hepatocyte growth factor (HGF), interferon (IFN)-α, IFN-ɣ, IL-1α, IL-1β, IL-2, IL-2R, IL-3, IL-4, IL-5, IL-6, IL-7, IL-8, IL-9, IL-10, IL-12p70, IL-13, IL-15, IL-16, IL-17A, IL-18, IL-20, IL-21, IL-22, IL-23, IL-27, IL-31, interferon γ-induced protein 10 kDa (IP-10), interferon-inducible T cell alpha chemoattractant (I-TAC), leukaemia inhibitory factor (LIF), MCP-1, MCP-2, MCP-3, macrophage colony-stimulating factor (M-CSF), macrophage-derived chemokine (MDC), macrophage migration inhibitory factor (MIF), monokine induced by gamma interferon (MIG), MIP-1α, MIP-1β, MIP-3α, matrix metalloproteinase-1 (MMP-1), nerve growth factor β (NGF-β), stem cell factor (SCF), stromal cell-derived factor-1α (SDF-1α), TNF-α, TNF-β, TNF-receptor (R)-2, tumour necrosis factor related apoptosis inducing ligand (TRAIL), thymic stromal lymphopoietin (TSLP), tumour necrosis factor-like weak inducer of apoptosis (TWEAK), and vascular endothelial growth factor-A (VEGF-A). At -80 °C stored AIS and sCeAD-nonAIS plasma sample aliquots were thawed on ice. Then, samples were centrifuged at 10,000 g for 10 min to pellet out lipid particulates. Afterwards, the plasma samples were immediately used. First, magnetic beads in a 96-well flat-bottom plate were washed and 25 µl of universal assay buffer along with 25 µl of undiluted plasma samples or a four-fold serial diluted standard was added into each well. The samples were incubated for 120 min at room temperature (RT) on a shaker at 500 rounds per minute, followed by washing. Next, 25 ml of detection antibody mixture was added to each well and incubated for 30 min at RT on a shaker, then washed twice. Afterwards, 50 µl of streptavidin–phycoerythrin solution was added, and the plates were placed on a shaker for another 30 min incubation at RT. Then, the plates were washed again, and beads were dissolved in 120 µl of reading buffer. After 5 min of incubation while being gently shaken at RT, fluorescence intensity was measured using Luminex MAGPIX instrument according to the manufacturer’s instructions (Software: xPonent 4.2 and ProcartaPlex Analyst 1.0). In samples exceeding the concentration of the highest standard, this concentration limit was used for further analysis. Only cytokines/chemokines with more than 10% of all baseline samples showing levels above the lowest standard were included in further analyses.

### Statistical analysis

Statistical analyses were performed using GraphPad Prism-10 (GraphPad Software Inc., La Jolla, California, United States) and SPSS-27 (IBM SPSS Statistics, Armonk, NY). All figures were created by the authors using GraphPad Prism-10.

The primary aim of the study was to compare the peripheral cytokine/chemokine profile in people after AIS classified by high-sensitivity CRP levels at admission, those AIS patients with unaltered peripheral inflammatory responses at admission, and people without stroke having sCeAD with local symptoms only. For overall multiple group comparisons, the non-parametric Kruskal–Wallis test was used. P-values were corrected for multiple (n = 65) comparisons using Bonferroni’s correction. Effect size coefficients η^2^ were calculated from the Kruskal–Wallis statistics using the formula $\eta 2=\frac{(H-k+1)}{(n-k)}$ (H = Kruskal–Wallis H value; k = number of groups; n = number of total observations) and classified as either small (0.01 to < 0.06), moderate (0.06 to < 0.14), or large (η^2^ ≥ 0.14) according to Cohen, 1988^30^. Dunn’s multiple comparisons tests were used to compare subgroups. The necessary sample size for the Kruskal–Wallis test (for 5% α and 80% power (1-ß)) was calculated using G*Power software based on F values calculated from η^2^ values (F = $\sqrt{\frac{{\eta }^{2}}{(1-{\eta }^{2})}}$). To detect a moderate effect size of F > 0.25 (corresponding to η^2^ > 0.06) it was determined to be > 159 (total sample size) and to detect a large effect size of F > 0.40 (corresponding to η^2^ > 0.14) it was determined to be > 66 (total sample size).

Secondary aims were (i) to perform subtype-stratified analyses to assess potential differences among various AIS aetiologies (using the non-parametric Kruskal–Wallis test). The necessary sample size was calculated as described above and was determined to be > 180 (total sample size) to detect a moderate effect size of F > 0.25 (corresponding to η^2^ > 0.06) or > 76 (total sample size) to detect a large effect size of F > 0.40 (corresponding to η^2^ > 0.14). Moreover, a multivariate linear regression model was used to predict the role of clinical (age, sex, ischaemic stroke, aetiology, NIHSS, and mRS score) and laboratory (CRP) parameters on baseline plasma levels of significantly altered analytes. Cytokine levels were log10 transformed to meet the assumption of the model, and results are shown as estimates b (with standard error of mean) and standardised estimates ß (with 95% confidence intervals).

(ii) To analyse the associations of altered cytokines/chemokines with demographic, clinical, and laboratory characteristics of the acute event (using non-parametric Spearman’s rank correlation test, Mann–Whitney *U* test, chi-square test, or Fisher’s exact test). Due to limited statistical power and the large number of statistical comparisons, we only show effect sizes, but no p-values. Effect size coefficients R were calculated from the Mann–Whitney U statistics using to the formula $R= \frac{z}{\sqrt{N}}$ (z = Mann–Whitney *U* z-value; N = total number of ranks). Calculated R and Spearman ρ effect sizes were classified as either small (-0.3 to -0.1, or 0.1 to 0.3), moderate (-0.5 to -0.3, or 0.3 to 0.5), or large (< -0.5 or > 0.5) according to Cohen, 1988^30^. Effect sizes were then grouped by k-means clustering (Pearson’s correlation) and are shown as a heat map.

(iii) To investigate the association of baseline cytokine/chemokine levels for functional outcome (by multivariate binary logistic regression analysis).

(iv) To examine if levels of cytokines and chemokines remain stable in follow-up samples in a subgroup of AIS patients up to twelve months and to analyse their association with AIS subtypes and outcome (using the non-parametric Friedman test or Kruskal–Wallis test with Dunn’s multiple comparison tests).

Statistical significance was defined as two-sided p-value < 0.05 after correction for multiple comparisons using Bonferroni’s correction.

### Ethics approval and consent to participate

The use of frozen samples from a biobank for this study was approved by the local ethics committees of the Medical University Innsbruck, Austria (ReSect study: EK#UN5072, 325/4.1, 29.03.2019^31^; STROKE-CARD study: EK# UN2013-0045 and is registered with Clinical-Trials.gov number NCT02156778 (03.01.2014)^23^). All patients or their legal representatives gave written informed consent to diagnostic procedures and biological sample storage for research purposes.

## Results

### Baseline characteristics of study participants

Baseline characteristics of patients after AIS classified according to their baseline CRP levels and characteristics of sCeAD-nonAIS patients are presented in Table 1. Among all AIS patients, 49 (28.3%) were classified as having baseline CRP levels of > 5 mg/l, and 124 (71.7%) had CRP levels ≤ 5 mg/l. Cerebrovascular risk factors of all stroke patients grouped according to the underlying aetiology are listed in Table 2, along with Table S1 of the Supplementary file 1.

**Table 1:** Demographics, clinical and routine blood parameters of people after acute ischaemic stroke and cervical artery dissection with local symptoms only.

	Stroke with CRP > 5 mg/l (n = 49)	Stroke with CRP ≤ 5 mg/l (n = 124)	sCeAD-nonAIS (n = 42)
Female:male, n (%)	20 (40.8):29 (59.2)	36 (29.0):88 (71.0)	19 (45.2):23 (54.8)
Median age [years]	48.8 (27.0–90.0)	53.0 (20.6–92.0)*	47.5 (29.3–66.6)
AIS aetiology, n (%)			
LAA	6 (12.3)	24 (19.4)	–
CE	8 (16.3)	26 (21.0)	–
SVO	3 (6.1)	12 (9.7)	–
sCeAD	32 (65.3)	62 (50.0)	–
Median CRP value [mg/l]	7.0 (5.2–31.5)^†^*	1.1 (0.0–5.0)*	2.8 (0.6–28.9)
Coagulation parameters			
INR	1.0 (0.9–1.9)^†^	1.0 (0.9–2.7)	1.0 (0.9–2.2)
PT [%]	101.0 (34.0–126.0)	99.0 (29.0–116.0)	97.5 (32.0–130.0)
Platelet level [G/l]	252.5 (123.0–562.0)^†^*	208.0 (63.0–660.0)	197.0 (69.0–572.0)
d-Dimer [µg/l]	190.0 (40.0–2150.0)	282.5 (66.0–4870.0)	222.0 (51.0–1258.0)
AT-III [%]	90.0 (71.0–120.0)^†^	85.0 (62.0–115.0)	88.5 (73.0–118.0)
Lipids			
Total cholesterol [mg/dl]	184.0 (117.0–311.0)	182.0 (90.0–303.0)	207.0 (112.0–300.0)
HDL-C [mg/dl]	51.5 (16.0–93.0)*	52.0 (25.0–139.0)*	57.5 (35.0–100.0)
LDL-C [mg/dl]	119.0 (60.0–203.0)	113.0 (36.0–224.0)	121.0 (46.0–217.0)
Triglyceride level [mg/dl]	108.0 (48.0–415.0)	103.0 (41.0–366.0)	96.5 (38.0–614.0)
Lipoprotein [mg/dl]	20.0 (20.0–239.2)	20.0 (20.0–378.2)	20.0 (20.0–238.7)
Fasting glucose [mg/dl]	90.5 (66.0–308.0)	91.0 (70.0–363.0)	88.5 (73.0–128.0)
HbA_1c_ [%]	5.5 (4.5–13.7)^†^*	5.4 (4.5–12.7)	5.3 (4.7–6.9)
Acute intervention			
Thrombolysis, n (%)	8 (16.3)	19 (15.3)	–
Thrombectomy, n (%)	0 (0)	4 (3.2)	–
NIHSS at baseline	3 (0–21)*	2 (0–28)*	–
NIHSS stroke severity groups at baseline			
Minor (NIHSS 0–5)	31 (63.3)	92 (74.2)	–
Moderate (NIHSS 6–15)	14 (28.6)	23 (18.6)	–
Severe (NIHSS ≥ 16)	3 (6.1)	8 (6.5)	–
mRS at baseline	3 (0–5)*	2 (0–5)*	–
mRS at last clinical follow-up^1^	0 (0–4)*	0.5 (0–4)	–
Good functional outcome at last clinical follow-up^1^ (mRS ≤ 2)^1^, n (%)	41 (83.7)	114 (91.9)	–
Unfavourable functional outcome at last clinical follow-up^1^ (mRS > 2)^1^, n (%)	8 (16.3)	10 (8.1)	–
Median time from baseline until last clinical mRS follow-up [years]^1^	5.1 (0.9–16.9)^†^	1.4 (0.8–20.4)*	–

Values are expressed as percentages or median (min–max).

*AIS* acute ischaemic stroke, *AT-III* antithrombin-III, *CE* cardioembolism, *CRP* C-reactive protein, *HbA1c* glycated haemoglobin (A1c), *HDL*-*C* high-density lipoprotein cholesterol, *HGF* hepatocyte-growth-factor, *INR* international normalised ratio, *IL* interleukin, *LAA* large artery atherosclerosis, *LDL-C* low-density lipoprotein cholesterol, *mRS* modified Rankin Scale, *NIHSS* National Institutes of Health Stroke Scale, *PT* prothrombin time, *sCeAD* spontaneous cervical artery dissection, *sCeAD*-*nonAIS* spontaneous cervical artery dissection with local symptoms only (i.e. no ischaemic stroke), *SDF-1α* stromal-cell-derived factor-1α, *SVO* small vessel occlusion.

^1^Cytokine levels were only measured at month three and twelve follow-up time point in a subgroup of AIS patients, not at the last clinical follow-up where the mRS was assessed. *p < 0.05 vs sCeAD-nonAIS, ^†^p < 0.05 vs stroke with CRP ≤ 5 mg/l patients.

**Table 2:** Baseline risk factors of people after ischaemic stroke and cervical artery dissection.

	Large artery atherosclerotic stroke (n = 30)	Cardioembolic stroke (n = 34)	Small vessel occlusion stroke (n = 15)	Cervical artery dissection (n = 136^1^)
BMI [kg/m^2^]	25.3 (21.3–33.3)	26.2 (17.3–39.1)	25.1 (20.6–35.0)	25.6 (17.4–35.4)
Diabetes mellitus, n (%)	6 (20.0)*	3 (8.8)	3 (20.0)*	2 (1.5)
Hypertension, n (%)	24 (80.0)*	30 (88.2)*	14 (93.3)*	46 (34.6)
Dyslipidaemia, n (%)	30 (100)*	27 (79.4)*	12 (80.0)*	60 (45.1)
Smoking, n (%)	8 (26.7)	5 (14.7)	2 (13.3)	42 (31.6)
CCA/ICA-stenosis > 50%, n (%)	19 (63.3)*	3 (8.8)*	0 (0)	0 (0)
Clinical history, n (%)				
Atrial fibrillation	0 (0)	26 (76.5)*	0 (0)	1 (0.7)
Myocardial infarction	3 (10.0)	6 (17.6)*	0 (0)	3 (2.3)
Heart intervention	1 (3.3)	9 (26.5)*	1 (6.7)	0 (0)
Peripheral artery disease	2 (6.7)	1 (2.9)	0 (0)	1 (0.8)
Cerebrovascular history	5 (16.7)*	4 (11.8)*	3 (20.0)*	0 (0)
TIA	3 (100)*	0 (0)	3 (100)*	0 (0)

Patients with cervical artery dissection leading to stroke (n = 94) and those with local symptoms only (n = 42) were grouped together as no differences between these two were found.

Values are expressed as percentages or median (min–max).

*BMI* body mass index, *CCA*/*ICA* common carotid artery/internal carotid artery, *TIA* transient ischaemic attack.

^1^Including 94 patients with cervical artery dissection leading to stroke and 42 patients having cervical artery dissection with local symptoms only; *p < 0.05 vs patients with cervical artery dissection using chi-square test or Fisher’s exact test.

### Altered cytokines/chemokines in AIS compared to sCeAD-nonAIS

**Figure 1:**
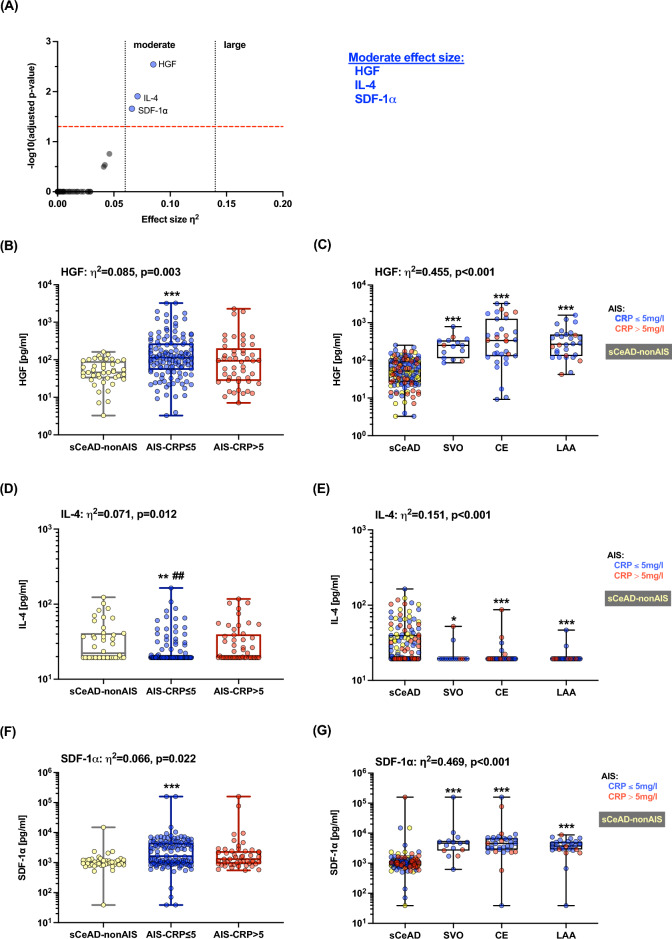
Altered plasma cytokine/chemokine concentrations in people with different ischaemic stroke (AIS) aetiologies and people with spontaneous cervical artery dissection with local symptoms only (sCeAD-nonAIS). (**A**) Volcano plot showing η^2^ effect sizes versus -log10 adjusted p-values. The significance threshold for p-values adjusted by Bonferroni’s correction for 65 comparisons is indicated by the red dashed line. Levels for moderate (η^2^ > 0.06) and large (η^2^ > 0.14) effect size are indicated by dotted lines. Plasma concentrations [pg/ml] of (**B**) HGF, (**D**) IL-4, and (**F**) SDF-1α in people with stroke and CRP levels > 5 mg/l, n = 49 or stroke with CRP levels ≤ 5 mg/l, n = 124 and sCeAD-nonAIS, n = 42. Plasma concentrations [pg/ml] of (**C**) HGF, (**E**) IL-4, and (**G**) SDF-1α in people with sCeAD (with and without AIS) and patients with different mechanism leading to stroke (small vessel occlusion (SVO) n = 15; cardio embolism (CE) n = 34; large artery atherosclerosis (LAA) n = 30). sCeAD-nonAIS patients (n = 42) are indicated in yellow. Multiple comparisons were performed using Kruskal–Wallis test with Dunn’s multiple comparison correction (compared to sCeAD (*) or AIS-CRP > 5 mg/l (^#^): ***p < 0.001, **/^##^p < 0.01, *p < 0.05). *CE* cardioembolic stroke, *HGF* hepatocyte-growth-factor, *IL* interleukin, *LAA* large artery atherosclerotic stroke, *SDF*-*1α* stromal-cell-derived factor-1α, *SVO* small-vessel occlusive stroke.

A comparison between the two stroke groups (baseline CRP levels ≤ 5 mg/l and CRP levels > 5 mg/l) and sCeAD-nonAIS revealed differences after Bonferroni’s correction for multiple comparisons for the following cytokines and chemokines: HGF (p = 0.00004, η^2^ = 0.085), IL-4 (p = 0.0002, η^2^ = 0.071), and SDF-1α (p = 0.0003, η^2^ = 0.066), with moderate effect sizes (Fig. 1). All other 62 cytokines/chemokines for which differences were not found between the AIS groups with different CRP levels and sCeAD-nonAIS are displayed in the Supplementary Table S2. HGF and SDF-1α plasma levels were lower in the sCeAD-nonAIS cohort compared to AIS with CRP levels ≤ 5 mg/l. In contrast, IL-4 plasma levels were higher in sCeAD-nonAIS. Moreover, only IL-4 levels differed between AIS patients with CRP levels of ≤ 5 mg/l or > 5 mg/l (Fig. 1).

**Figure 2:**
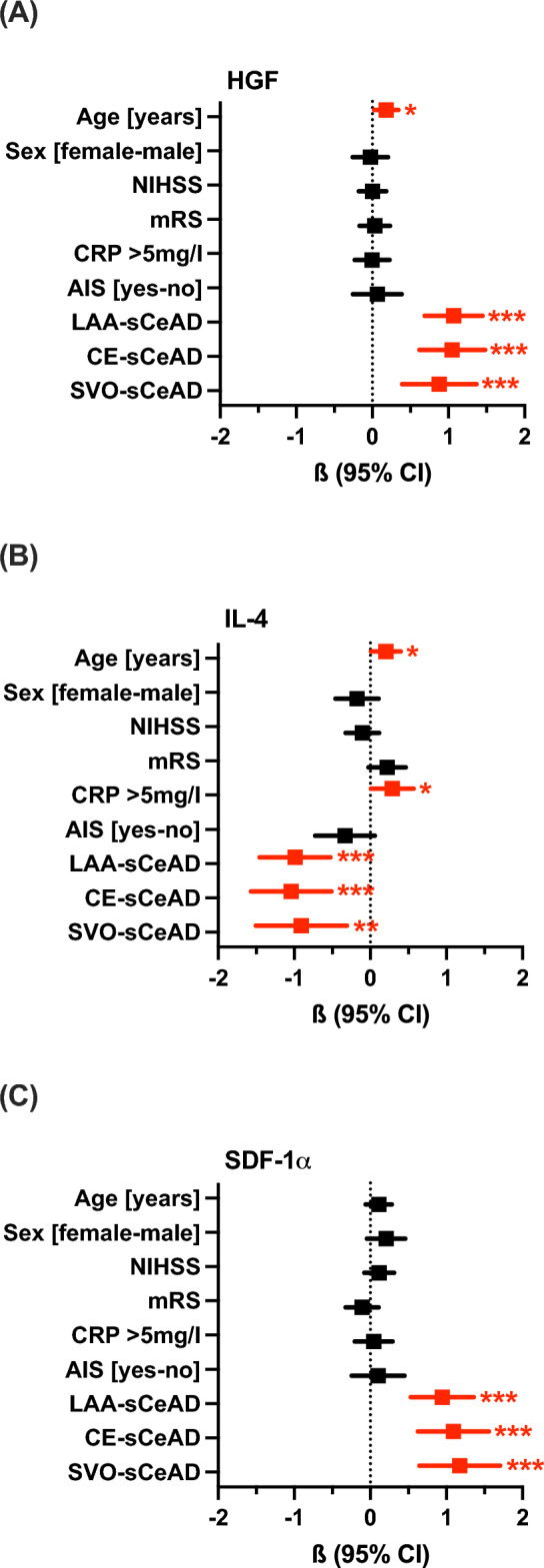
Multivariate linear regression model to predict the role of clinical and laboratory parameters on baseline plasma levels of (**A**) HGF, (**B**) IL-4, and (**C**) SDF-1α. Results are shown as standardised estimates ß with 95% CI (confidence intervals). All important predictors are shown in red, and asterisks indicate the level of significance (***p < 0.001; **p < 0.01; *p < 0.05). HGF, IL-4, and SDF-1α levels were log10 transformed to meet the assumptions of the model. Model fit: (**A**) R = 0.671, R^2^ = 0.450, F = 18.3, p < 0.001, collinearity: VIF < 2.0, autocorrelation: R = -0.02. (**B**) R = 0.417, R^2^ = 0.174, F = 4.7, p < 0.001, collinearity: VIF < 2.0, autocorrelation: R = 0.14. (**C**) R = 0.595, R^2^ = 0.354, F = 12.3, p < 0.001, collinearity: VIF < 2.0, autocorrelation: R = 0.08. More details are provided in Tables S3–S5. *AIS* acute ischaemic stroke, *CE* cardioembolic stroke, *CRP* C-reactive protein, *HGF* hepatocyte-growth-factor, *IL* interleukin, *LAA* large artery atherosclerotic stroke, *mRS* modified Rankin Scale, *NIHSS* National Institutes of Health Stroke Scale, *sCeAD* spontaneous cervical artery dissection leading to stroke or local symptoms only, *SDF*-*1α* stromal-cell-derived factor-1α, *SVO* small vessel occlusive stroke.

The comparison of underlying causes for AIS showed that sCeAD was associated with lower plasma levels of HGF (p < 0.001, η^2^ = 0.455, large effect size) and SDF-1α (p < 0.001, η^2^ = 0.469, large effect size) compared to AIS due to SVO, CE, and LAA (Fig. 1). Only for IL-4 (p < 0.001, η^2^ = 0.151, large effect size) were higher levels found in people with sCeAD compared to other stroke aetiology groups. Levels of these cytokines were comparable between sCeAD-AIS and sCeAD-nonAIS cohorts (Supplementary Fig. S1). Since the effect sizes of these comparisons were much larger than those of the initial comparisons, we performed a multivariate linear regression analysis for HGF, IL-4, and SDF-1α adjusted for age, sex, CRP levels > 5 mg/l, NIHSS, and mRS score, AIS, and aetiology (Fig. 2 and Supplementary Tables S3–S5). Results clearly indicate that the strongest predictor of HGF, IL-4, and SDF-1α levels was aetiology, followed by age for HGF and IL-4, and CRP levels > 5 mg/l for IL-4.

**Figure 3:**
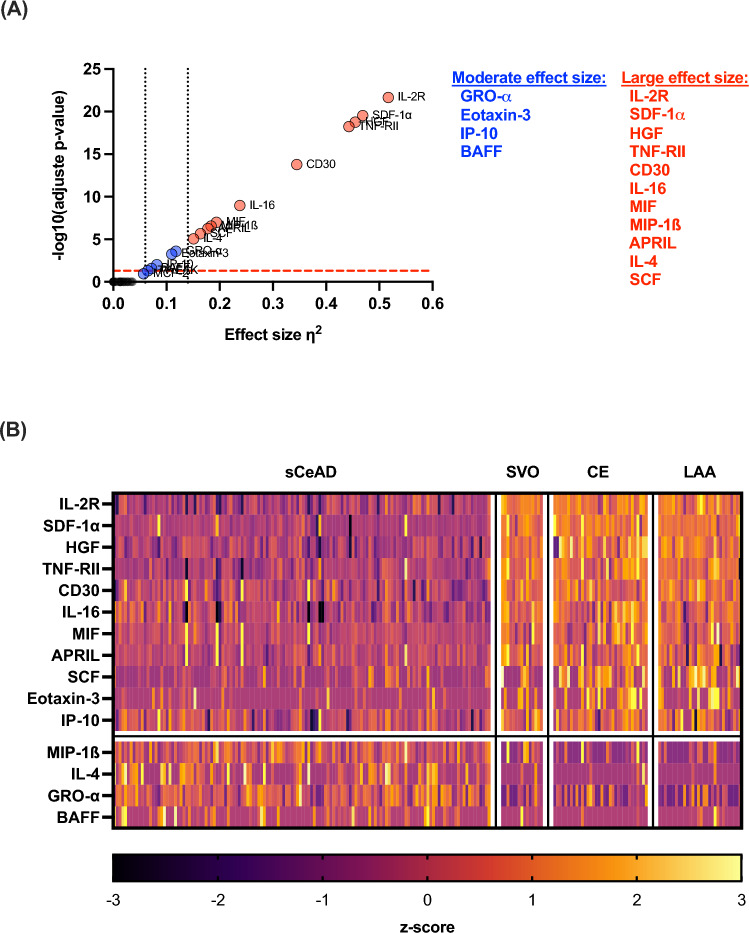
Altered plasma cytokine/chemokine concentrations in people with different stroke aetiologies. (**A**) Volcano plot showing η^2^ effect sizes versus -log10 adjusted p-values. The significance threshold for p-values adjusted by Bonferroni’s correction for 65 comparisons is indicated by the red dashed line. Levels for moderate (η^2^ > 0.06) and large (η^2^ > 0.14) effect size are indicated by dotted lines. (**B**) Heatmap of altered analytes shown as standardised values (z-scores) of log10 transformed cytokine values grouped according to their aetiology. Negative z-scores (decreased levels of analytes) are shown by dark colours, positive z-scores (increased levels of analytes) are shown by light colours. The heatmap was created by the authors using GraphPad Prism-10 (GraphPad Software Inc., La Jolla, California, United States; https://www.graphpad.com). *APRIL* a proliferation-inducing ligand, *BAFF* B-cell activating factor, *CD30* TNF receptor superfamily member 8, *CE* cardioembolic stroke, *GRO*-*α* growth regulated oncogene-α, *HGF* hepatocyte-growth-factor, *IL* interleukin, *IL-2R* interleukin-2 receptor, *IP*-*10* interferon γ-induced protein 10 kDa, *LAA* large artery atherosclerotic stroke, *MIF* macrophage migration inhibitory factor, *MIP*-*1β* macrophage inflammatory protein-1β, *sCeAD* spontaneous cervical artery dissection leading to stroke, *SCF* stem cell factor, *SDF*-*1α* stromal-cell-derived factor-1α, *SVO* small vessel occlusive stroke, *TNF*-*RII* tumour necrosis factor receptor 2.

**Figure 4:**
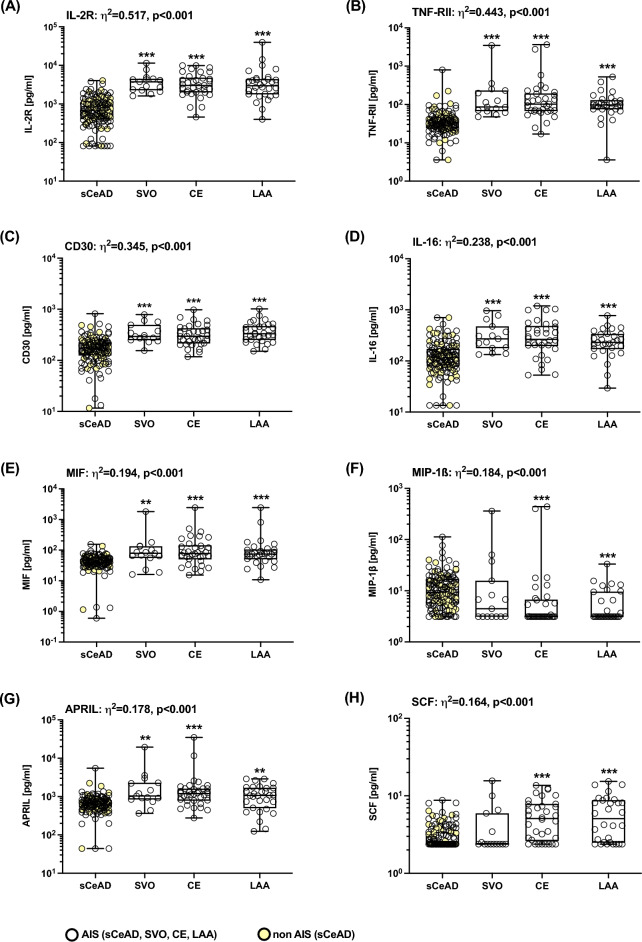
Altered plasma cytokine/chemokine concentrations in people with different stroke aetiologies. Plasma levels of (**A**) IL-2R, (**B**) TNF-RII, (**C**) CD30, (**D**) IL-16, (**E**) MIF, (**F**) MIP-1β, (**G**) APRIL, and (**H**) SCF were grouped based on the underlying mechanisms: spontaneous cervical artery dissection (sCeAD) with AIS (n = 94) or local symptoms only (n = 42, shown in yellow); small vessel occlusive stroke (SVO) n = 15; cardio embolic stroke (CE) n = 34; large artery atherosclerotic stroke (LAA) n = 30. Group comparisons were performed using Kruskal–Wallis test with Dunn’s multiple comparison tests (difference to sCeAD: ***p < 0.001; **p < 0.01; *p < 0.05). *AIS* acute ischaemic stroke, *APRIL* a proliferation-inducing ligand, *CD30* TNF receptor superfamily member 8, *IL* interleukin, *IL*-*2R* interleukin 2 receptor, *MIF* macrophage migration inhibitory factor, *MIP*-*1β* macrophage inflammatory protein-1β, *SCF* stem cell factor, *TNF*-*RII* tumour necrosis factor receptor 2.

Due to this result, we next compared the levels of all analytes among the different aetiologies. We detected differences after Bonferroni’s correction for the following cytokines and chemokines: HGF, SDF-1α, IL-2R, CD30, TNF-RII, IL-16, MIF, MIP-1β, APRIL, SCF, and IL-4 (all large effect sizes); and GROα, Eotaxin-3, IP-10, and BAFF (all moderate effect sizes). However, since the necessary sample size was determined to be > 180 (for 4 groups, 45/group) to detect a moderate effect size of F > 0.25 (corresponding to η^2^ > 0.06) and the sizes of three groups were smaller (LAA, n = 30; CE, n = 34; SVO, n = 15), we only considered results with a large effect size F ≥ 0.40 (corresponding to η^2^ ≥ 0.14). Therefore, only the results of HGF, SDF-1α, IL-2R, CD30, TNF-RII, IL-16, MIF, MIP-1β, APRIL, SCF, and IL-4 are statistically significant with sufficient power (Figs. 3, 4, Supplementary Table S6). Finally, we also compared the pooled LAA, CE, and SVO group with sCeAD and confirmed our findings, whereas there were no differences between the two sCeAD subgroups (sCeAD-nonAIS versus sCeAD-AIS). Increased CRP levels > 5 mg/l had no impact on levels of cytokines and chemokines (Supplementary Fig. S1).

### Differences in HGF, SDF-1α, IL-2R, CD30, TNF-RII, IL-16, MIF, MIP-1β, APRIL, SCF, and IL-4 levels according to demographic, clinical and laboratory parameters at admission

**Figure 5:**
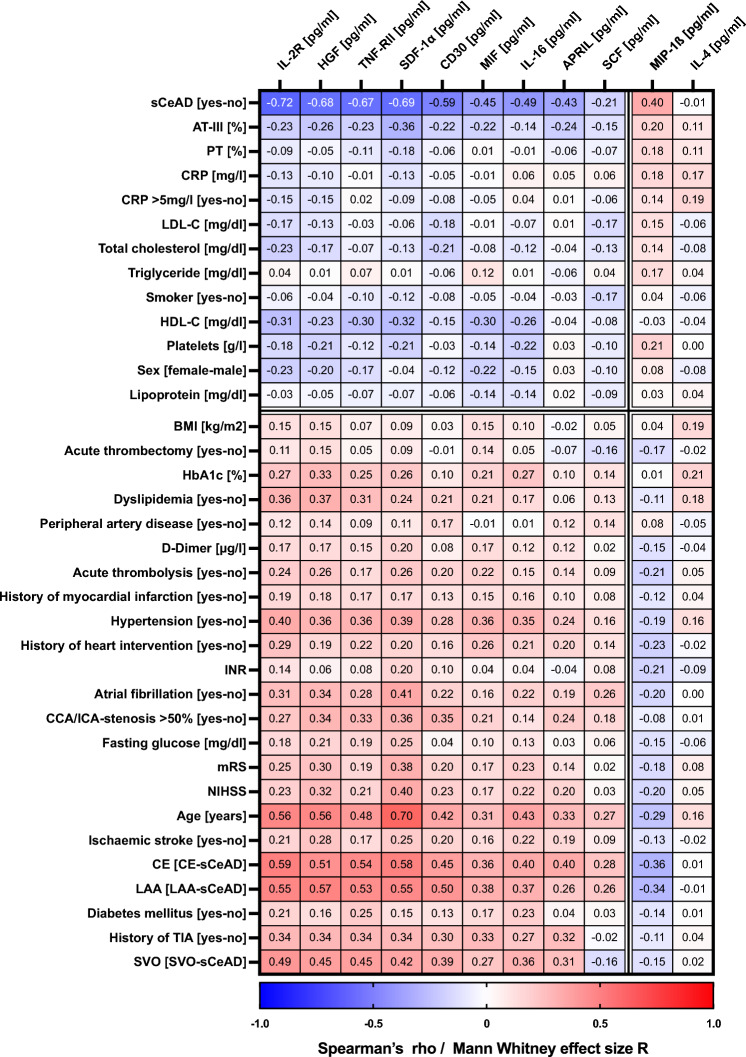
Heatmap of Spearman’s correlation coefficients for continuous clinical and laboratory characteristics or Mann–Whitney U test effect sizes for dichotomous characteristics in comparison to baseline plasma levels of IL-2R, HGF, TNF-RII, SDF-1α, CD30, MIF, IL-16, APRIL, SCF, MIP-1β, and IL-4. Data are depicted in colours ranging from blue to red, indicating a negative to positive effect size R or Spearman’s ρ grouped by k-means clustering. Samples from all 215 study participants (AIS and sCeAD-nonAIS) at baseline were included in this figure. The heatmap was created by the authors using GraphPad Prism-10 (GraphPad Software Inc., La Jolla, California, United States; https://www.graphpad.com). *APRIL* a proliferation-inducing ligand, *AT*-*III* antithrombin-III, *CCA*/*ICA* common carotid artery/internal carotid artery, *CD30* TNF receptor superfamily member 8, *CE* cardioembolic stroke, *CRP* C-reactive protein, *HbA1c* glycated haemoglobin A1c, *HDL*-*C* high-density lipoprotein cholesterol, *HGF* hepatocyte-growth-factor, *IL* interleukin, *IL*-*2R* interleukin 2 receptor, *INR* international normalised ratio, *LAA* large artery atherosclerotic stroke, *LDL*-*C* low-density lipoprotein cholesterol, *MIF* macrophage migration inhibitory factor, *MIP*-*1β* macrophage inflammatory protein-1β, *mRS* modified Rankin Scale, *NIHSS* National Institutes of Health Stroke Scale, *PT* prothrombin time, *sCeAD* spontaneous cervical artery dissection, *SCF* stem cell factor, *SDF*-*1α* stromal-cell-derived factor-1α, *SVO* small vessel occlusive stroke, *TIA* transient ischaemic attack, *TNF*-RII tumour necrosis factor receptor 2.

Next, we analysed the associations of these altered cytokines/chemokines (HGF, SDF-1α, IL-2R, CD30, TNF-RII, IL-16, MIF, MIP-1β, APRIL, SCF, IL-4) with demographic, clinical, and laboratory characteristics of the acute event in the entire study cohort. The heatmap in Fig. 5 shows the effects sizes (R and ρ) of these comparisons/correlations according to hierarchical clustering. We could identify two groups of analytes with differential associations: (i) HGF, SDF-1α, IL-2R, CD30, TNF-RII, IL-16, MIF, APRIL, and SCF were negatively associated with sCeAD, CRP, lipids, platelets, and female sex, and positively associated with AIS, LAA, CE, SVO, age, diabetes, dyslipidaemia, hypertension, HbA1c, atrial fibrillation, and a positive history of a transient ischaemic attack.

In contrast, MIP-1β and IL-4 showed a completely different picture with a positive association with sCeAD, CRP, and many coagulation parameters. Furthermore, especially MIP-1β showed a negative association with age, CE, LAA, SVO, INR, and hypertension among many more.

### Association of baseline cytokine/chemokine levels with functional outcome

We then analysed whether baseline levels of HGF, SDF-1α, IL-2R, CD30, TNF-RII, IL-16, MIF, MIP-1β, APRIL, SCF, and IL-4 predicted functional outcome (mRS 0–2 or mRS 3–5) in a multivariate binary logistic regression model adjusted for sex, CRP levels, NIHSS, and age at admission. Although baseline levels of HGF, SDF-1α, IL-2R, CD30, TNF-RII, IL-16, MIF, and APRIL were higher in AIS patients with mRS 3–5, in the multivariate model, only higher age and higher NIHSS assessed in the acute phase were associated with mRS > 2 (Supplementary Table S7).

### Stability of plasma cytokine/chemokine levels after month three and month twelve of follow-up in a subgroup of AIS patients

Plasma cytokine/chemokine levels were measured in a subgroup of people with AIS at a median of 3.3 (range 2.3–4.3) months, and for the last follow-up measurement at a median of 12.6 (range 11.1–14.2) months after the acute event. This subgroup included 30 patients with stroke due to LAA, 34 with CE stroke, and 15 with SVO stroke. Clinical and laboratory characteristics at these follow-up visits are shown in Supplementary Table S8 and Fig. S2.

A decrease in plasma levels comparing baseline and both follow-up time points was observed for HGF, and these plasma levels remained stable between three-month and twelve-month follow-up time points. Similarly, TNF-RII levels decreased three months after the acute event, while plasma CD30 levels increased during follow-up. Statistical testing to assess differences between stroke aetiology groups regarding baseline and follow-up cytokine/chemokine levels was not performed due to the limited sample size.

## Discussion

Our investigation into the systemic inflammatory response following AIS revealed distinct plasma concentrations of HGF, SDF-1α, and IL-4 compared to individuals with sCeAD-nonAIS. Our study highlighted unique inflammatory profiles between sCeAD and other stroke aetiologies, though no differences were found between sCeAD with or without stroke. The comparison of all 65 cytokines and chemokines among the four aetiology groups (sCeAD, LAA, CE, and SVO) revealed differences for eleven analytes. Plasma levels of HGF, SDF-1α, IL-2R, CD30, TNF-RII, IL-16, MIF, APRIL, and SCF were lower in sCeAD than in LAA, CE or SVO, but IL-4 and MIP-1β levels were higher in sCeAD. Elevated CRP levels were not associated with these cytokines and chemokines. In summary, our results indicate substantial differences in the inflammatory response between dissection (with or without stroke) and other stroke aetiologies (LAA, CE, and SVO).

Many of the proteins showing differences in our study play established roles in stroke or cardiovascular diseases. HGF, for instance, is recognised as an endothelium-specific growth factor with diverse beneficial functions, including anti-inflammatory, anti-fibrotic, and pro-angiogenetic properties^32^. It is primarily released in response to endothelial injury, leading to elevated levels observed in cardiovascular conditions such as atherosclerosis^33^, acute myocardial infarction^34^, or AIS^35^. Previous research has linked higher HGF levels with poor outcomes in these diseases^36,37^, likely due to increased endothelial release. Interestingly, our study revealed a decrease in HGF levels three months after the acute stroke event, suggesting its involvement not only in the inflammatory response to stroke but also in reflecting the response to cerebral ischaemia itself during the acute phase. In contrast to HGF, levels of most other cytokines/chemokines remained elevated up to one year, indicating a more generalised inflammatory response, as ischaemia itself would not be expected to drive inflammatory cytokine and chemokine levels to a similar extent after twelve months. This is consistent with previous findings where upregulated proteins during the acute phase of stroke remained elevated in follow-up measurements^38^.

SDF-1α is produced by activated astrocytes, microglia, and vascular endothelial cells. It plays a crucial role in recruiting monocytes locally to the injured brain region after ischaemic stroke^39–41^ and mobilises endothelial progenitor cells, thus promoting angiogenesis^42^. Elevated SDF-1α levels have been observed in individuals with cardiovascular risk factors^43,44^, and plasma levels have been linked to adverse cardiovascular outcomes in people with coronary artery disease^45^. Similarly, elevated levels of IL-2R have been reported in coronary artery atherosclerosis^46^ and associated with various cardiovascular risk factors, including diabetes mellitus and hypertension^47^, as confirmed in our study.

Members of the TNF receptor superfamily such as CD30, TNF-RII, and APRIL, expressed on various immune cells^48,49^ were found to be upregulated in the blood of ischaemic stroke patients in the sub-acute phase^50–52^. APRIL was shown to protect against atherosclerosis by binding to heparin-sulphate proteoglycans in mice, and decreased serum levels are associated with long-term cardiovascular mortality in individuals with atherosclerosis^53^. IL-16, released by activated CD8+ T cells, contributes to the inflammatory response following ischaemic stroke. It activates CD4+ T cells, monocytes, macrophages, and dendritic cells, leading to the expression of various inflammatory cytokines. This process drives secondary brain damage^19,54–56^. MIF, a pleiotropic inflammatory mediator with chemokine-like functions, promotes leukocyte migration to inflammatory sites, including atherosclerosis^57^. MIF is not only produced by monocytes, macrophages, B- or T-cells, but also by endothelial and epithelial cells^58^.

SCF, produced by bone marrow stromal cells, induces neuroproliferation, reduces infarct size, and improves functional outcome in ischaemic stroke models^59,60^.

Distinct findings of higher plasma IL-4 and MIP-1β levels were observed in sCeAD patients compared to other stroke aetiologies. IL-4 has an anti-inflammatory role and is a critical regulator of M2 polarization. Following its secretion by neurons in response to ischaemia, it stimulates microglial phagocytosis and enables clearance of apoptotic neurons. Consequently, it promotes long-term recovery of microglia/macrophages^61^. MIP-1β, a chemokine secreted by various vascular and hematopoietic cells^62^, is linked to atherosclerosis^63^. Elevated blood levels of MIP-1β are reported in people with this condition^64^. However, such elevations were not observed in cohorts of stroke patients^21^, warranting additional investigation into underlying mechanisms regarding its differential expression in distinct cardiovascular pathologies. IL-4, along with MIP-1β, was associated with distinct clinical parameters, showing mostly positive Spearman’s correlation values with platelet count, PT, AT-III, or CRP in our study. Interleukins play a unique role in megakaryopoiesis, thrombopoiesis, and platelet function, with the majority having positive effects. However, a subset such as IL-4 or IL-1α has been reported to possess inhibitory properties on megakaryocyte differentiation, thereby inhibiting platelet production^65–68^. This contradicts the findings of our study and warrants further investigation. This includes a more comprehensive characterization of the study participants, particularly regarding haematological disorders that may have influenced these outcomes.

Experimental studies and clinical trials using above-described cytokines and chemokines have shown promising results in ischaemia treatment. Preclinical studies on rats or mice demonstrated the positive effect of HGF on protecting blood–brain barrier integrity, reducing infarct volume, and improving functional recovery after stroke^69–72^. Clinical trials testing HGF in peripheral or coronary artery ischaemia proved good tolerance^73–75^. However, to our knowledge, such trials for ischaemic stroke have not been conducted yet. Other therapeutic approaches involving cytokines and chemokines have also yielded promising results, especially in animal models^76–79^.

In our study cohort, individuals after sCeAD, whether experiencing a stroke or only local symptoms, had the lowest levels of cytokines compared to other stroke aetiology groups during the acute phase. This is an important finding, as it distinguishes our study from previous research. While another research group examined a similar panel of cytokines and chemokines in the blood of AIS patients, they neither conducted a comparative analysis across different AIS aetiology groups nor included such a substantial cohort of patients experiencing AIS due to sCeAD, including those presenting solely with local symptoms^21^. However, another study, albeit with a smaller sample size of 29 patients diagnosed with sCeAD leading to AIS, reported comparable findings of decreased cytokine levels in this aetiology of AIS compared to other causes^38^.

Our results also indicate that only higher age and higher NIHSS score, but not levels of any cytokine/chemokine, were associated with mRS > 2 after twelve months, indicating unfavourable outcomes.

There are several limitations to this study. Firstly, it is retrospective and includes a relatively small number of AIS patients. Although the sample size was sufficient to detect a moderate effect size for the primary aim, unequal group sizes based on CRP levels and TOAST aetiologies limit the statistical power. To address this, we used Bonferroni’s correction for multiple comparisons and focused on cytokines and chemokines with large effect sizes. However, the small sample size for most subgroup analyses, especially when classifying AIS patients by mRS scores (≤ 2 or > 2), further limits statistical power. We hypothesize that more cytokine/chemokine levels might differ between groups, particularly when comparing stroke due to sCeAD and other aetiologies. Therefore, larger studies including more AIS patients and individuals with sCeAD are needed to confirm our results and establish causality between cytokine levels and clinical factors.

Secondly, the retrospective design and inclusion of stroke patients from two different trials (STROKE-CARD trial, ReSect-study) resulted in differing clinical data sets and some missing clinical information, potentially introducing reporting bias. The time span between the onset of AIS or sCeAD symptoms and blood sampling for cytokine/chemokine assessment varied and was not standardised, as blood sampling was always performed at 8 am.

Moreover, this study does not accurately reflect the natural distribution of ischaemic stroke causes. In this study, patients with sCeAD-AIS constitute the majority, whereas patients with CE, which typically represents a major aetiology in AIS, are underrepresented. Since sCeAD accounts for approximately 1–2% of stroke patients^80^, its prevalence in this study is disproportionate. This also affects the median age of participants, as sCeAD leading to AIS or local symptoms predominantly occurs in young and middle-aged adults, whereas AIS due to other aetiologies commonly affects older people^81^. The selection of a group of people with sCeAD-nonAIS might impact the altered cytokine profile observed. While some epidemiological observations suggest a link between inflammation and sCeAD, particularly following infections^82–84^, the exact underlying pathophysiological mechanisms remain unclear. Reported infections include respiratory and urinary tract infections, and gastroenteritis, typically appearing within four weeks before the sCeAD event^85^. Notably, these infections often present with mild symptoms and usually resolve before hospitalization^86^, so we speculate they may not influence the cytokine/chemokine profile observed post-sCeAD event in our study.

We categorised participants into CRP groups of ≤ 5 mg/l or > 5 mg/l. It is crucial to acknowledge that CRP, as an acute-phase protein, is a nonspecific indicator of inflammation. Elevated CRP levels can result from infections^87^, tissue damage associated with atherosclerosis or ischaemic stroke^88^, chronic diseases^89^, or autoimmune disorders^90,91^. Therefore, there is a possibility of misclassifying AIS patient into the > 5 mg/l group if elevated CRP levels did not originate from the acute stroke event.

Finally, we evaluated long-term functional outcomes at twelve months and at the last clinical follow-up visit, with a median of 5.1 years post-stroke in the > 5 mg/l CRP group and 1.4 years in the ≤ 5 mg/l CRP group using the mRS score^92^. Unfortunately, neuroimaging scans were not included to investigate potential associations between infarct volume and baseline cytokine/chemokine levels. Ischaemic lesion volume has been suggested as a surrogate imaging biomarker in the early clinical phase of AIS to predict functional outcomes^93^. Previous studies have established an independent relationship between ischaemic lesion volume and functional outcome^94^, with a strong correlation between ischaemic stroke infarct volume on follow-up CT and MRI scans and the mRS score^95^. However, these findings are influenced by factors such as ischaemic lesion location and the timing of lesion volume measurement or mRS assessment^96^. Furthermore, non-modifiable factors like older age, stroke severity, or pre-stroke dependency, as well as complications during hospitalization, including pneumonia, increased intracranial pressure, or cerebral oedema, affect outcomes after three months^97,98^. Unfortunately, we did not assess early in-hospital complications in our study cohort.

Strengths of the current study are the inclusion of well-characterised ischaemic stroke patients, with particular emphasis on a sizable cohort of sCeAD-AIS patients. Importantly, we directly compared various stroke aetiologies, enabling us to find differences in the profiles of numerous inflammation-associated proteins between AIS patients and additionally those experiencing only local symptoms of sCeAD. Moreover, the standardised blood-sampling at three and twelve months post-event enabled the acquisition of valuable long-term follow-up data on cytokine and chemokine levels. This aspect greatly contributes to our understanding of the dynamics of these inflammatory mediators in the aftermath of AIS.

## Conclusions

Measuring 65 plasma cytokines and chemokines revealed that individuals with stroke due to LAA, CE, and SVO have a stronger inflammatory response than those with sCeAD, particularly for the analytes HGF, SDF-1α, IL-2R, CD30, TNF-RII, IL-16, MIF, APRIL, and SCF.

### Supplementary Information

Supplementary Information.

## Data Availability

The datasets used and/or analysed during the current study are available from the corresponding author on reasonable request.

## Abbreviations

AIS: Acute ischaemic stroke
APRIL: A proliferation-inducing ligand
AT-III: Antithrombin-III
BAFF: B-cell activation factor
BLC: B-lymphocyte chemoattractant
CCA/ICA: Common carotid artery/internal carotid artery
CD30: TNF receptor superfamily member 8
CD40L: CD40-ligand
CE: Cardioembolism
CRP: C-reactive protein
ENA-78: Epithelial neutrophil-activating peptide-78
FGF: Fibroblast-growth factor
G-CSF: Granulocyte colony-stimulating factor
GM-CSF: Granulocyte–macrophage colony-stimulating factor
GRO: Growth-regulated oncogene
HbA1c: Glycated haemoglobin A1c
HDL-C: High-density lipoprotein cholesterol
HGF: Hepatocyte growth factor
IFN: Interferon
IL: Interleukin
IL-2R: Interleukin 2 receptor
INR: International normalised ratio
IP: Interferon-ɣ-induced protein
I-TAC: Interferon-inducible T-cell α-chemoattractant
LAA: Large artery atherosclerosis
LDL-C: Low density lipoprotein cholesterol
LIF: Leukaemia inhibitory factor
MCP: Monocyte chemoattractant protein
MCRP: Monocyte chemoattractant protein
M-CSF: Macrophage colony-stimulating factor
MDC: Macrophage-derived chemokine
MIF: Macrophage migration inhibitory factor
MIG: Monokine induced by interferon-ɣ
MIP: Macrophage inflammatory protein
MMP: Matrix metalloproteinase
mRS: Modified Rankin Scale
NGF: Nerve growth factor
NIHSS: National Institutes of Health Stroke Scale
PT: Prothrombin time
sCeAD: Spontaneous cervical artery dissection
sCeAD-nonAIS: Spontaneous cervical artery dissection with local symptoms only
SCF: Stem-cell factor
SDF-1α: Stromal cell-derived factor-1α
SVO: Small vessel occlusion
TIA: Transient ischaemic attack
TNF: Tumour necrosis factor
TRAIL: TNF-related apoptosis-inducing ligand
TSLP: Thymic stromal lymphopoietin
TWEAK: Tumour necrosis factor-like weak inducer of apoptosis
VEGF: Vascular endothelial growth factor
