# Supplementary material for: Peripheral inflammatory response in people after acute ischaemic stroke and isolated spontaneous cervical artery dissection
Source: Sci Rep. 2024 May 27;14:12063. doi: 10.1038/s41598-024-62557-3 (PMC11130263; doi:10.1038/s41598-024-62557-3)
Supplement: Supplementary file 1 — Supplementary Information. [file 41598_2024_62557_MOESM1_ESM.pdf]

SUPPLEMENTARY FILE 1

Supplementary information

Peripheral inflammatory response in people after acute ischaemic stroke and isolated spontaneous cervical artery dissection

Angelika Bauer[1,2,3], Christian Boehme[1], Lukas Mayer-Suess[1], Dagmar Rudzki[1,2], Michael Knoflach[1,2], Stefan Kiechl[1,2], Markus Reindl[1]

[1] Clinical Department of Neurology, Medical University of Innsbruck, Innsbruck, Austria

[2] VASCage Research Centre on Vascular Ageing and Stroke, Innsbruck, Austria

[3] Institute of Hygiene and Medical Microbiology, Medical University of Innsbruck, Innsbruck, Austria

Corresponding author: Markus Reindl

Clinical Department of Neurology, Medical University of Innsbruck, Innsbruck, Austria

E-Mail: markus.reindl@i-med.ac.at

Supplementary methods

For our study we have used plasma samples stored in a biobank from two previously performed studies, the STROKE-Card trial and the ReSect study.

STROKE-CARD trial: This study enrolled patients aged ≥18 years who were hospitalised due to acute ischaemic stroke (or transient ischaemic attack, however, these patients were not included in our study). Enrolment occurred during the acute hospital stay. Only those with ischaemic stroke due to a cardioembolic event, large-artery atherosclerosis, or small-artery occlusion were included. Those with stroke due to uncommon or undetermined causes were excluded. Additional exclusion criteria comprised malignancies or other severe diseases with a life expectancy of less than one year, drug addiction, severe alcohol abuse, residence outside the catchment area, or permanent severe disability and low perspectives for successful rehabilitation (indicated by a mRS score of 5 at hospital discharge).

Outpatient appointment was scheduled at three and twelve months post-event, including neurological evaluations to assess clinical and functional outcomes (mRS, NIHSS, Barthel Index), routine laboratory examination, evaluation of recurrent cardiovascular events, hospital stays, procedures, falls, fractures, incident diseases, morbidities, current medication, adherence to prescribed drugs, and risk factor control. Additionally, ischaemic stroke aetiology was reevaluated according to TOAST criteria during the three month appointment (1).

Further details about other assessed endpoints of the STROKE-CARD trial, which were not included in this study, are provided elsewhere (2).

ReSect study: The ReSect study included people treated at the Department of Neurology at the Medical University of Innsbruck, meeting the following criteria: (i) confirmed diagnosis of CeAD via MRI documentation of an intramural hematoma in T1-weighted fat-saturated MRI imaging of cervical vessels, (ii) spontaneous occurrence or following minimal trauma (i.e. hyperextension, rotation, lateroversion of the neck), and (iii) extradural origin of CeAD (extension to the V4-segment was not an exclusion criterion). Exclusion criteria comprised timely association with high impact trauma, signs of external or internal injury other than CeAD, and sole intracranial artery dissection. Additionally, individuals with clinical signs of hereditary monogenetic connective tissue disease were excluded.

The ReSect study was designed as a long-term follow-up of sCeAD patients, with visits scheduled at least one year post sCeAD event. These study-specific visits included physical examination, psychosocial outcome assessment through questionnaires, and detailed history-taking by experienced stroke neurologists. NIHSS and mRS scores were assessed at hospital admission, discharge, and follow-up visits. For the current study, only patients who underwent blood drawing at baseline of the sCeAD event were included.

Further details on patient recruitment and selection characteristics were published elsewhere (3, 4).

Supplementary References

1. Adams HP, JR, Bendixen BH, Kappelle LJ, Biller J, Love BB, Gordon DL et al. Classification of subtype of acute ischaemic stroke. Definitions for use in a multicenter clinical trial. TOAST. Trial of Org 10172 in Acute Stroke Treatment. Stroke 1993; 24(1):35–41.

2. Toell T, Boehme C, Mayer L, Krebs S, Lang C, Willeit K et al. Pragmatic trial of multifaceted intervention (STROKE-CARD care) to reduce cardiovascular risk and improve quality-of-life after ischaemic stroke and transient ischaemic attack -study protocol. BMC Neurol 2018; 18(1):187.

3. Mayer-Suess L, Pechlaner R, Barallobre-Barreiro J, Boehme C, Toell T, Lynch M et al. Extracellular matrix protein signature of recurrent spontaneous cervical artery dissection. Neurology 2020; 95(15):e2047-e2055.

4. Mayer L, Boehme C, Toell T, Dejakum B, Willeit J, Schmidauer C et al. Local Signs and Symptoms in Spontaneous Cervical Artery Dissection: A Single Centre Cohort Study. J Stroke 2019; 21(1):112–5.

Table S1. Baseline risk factors of people with cervical artery dissection leading to ischaemic stroke or local symptoms only.

	**Cervical artery dissection leading to stroke (n=94)**	**Cervical artery dissection with local symptoms (n=42)**
BMI [kg/m^2]	26.0 (17.4-35.1)*	23.4 (17.6-35.3)
Diabetes mellitus, n (%)	1 (1.1)	1 (2.4)
Hypertension, n (%)	33 (35.9)	13 (31.7)
Dyslipidaemia, n (%)	45 (48.9)	15 (36.6)
Smoking, n (%)	29 (31.5)	13 (31.7)
CCA/ICA-stenosis >50%, n (%)	0 (0)	0 (0)
Clinical history, n (%)		
Atrial fibrillation	1 (1.1)	0 (0)
Myocardial infarction	1 (1.1)	2 (4.9)
Heart intervention	0 (0)	0 (0)
Peripheral artery disease	1 (1.1)	0 (0)
Cerebrovascular history	0 (0)	0 (0)
TIA	0 (0)	0 (0)

Values are expressed as percentages or median (min-max); * p<0.05 vs patients with cervical artery dissection having local symptoms only. Abbreviations: BMI, body mass index; CCA/ICA, common carotid artery/internal carotid artery; TIA, transient ischaemic attack.

Table S2. Plasma concentrations of 65 cytokines, chemokines, and related molecules in individuals after acute ischaemic stroke, grouped based on CRP levels at the acute event, and individuals with spontaneous cervical artery dissection presenting with local symptoms only.

	**Spontaneous cervical artery dissection without stroke (n=42)**			**Stroke with CRP >5 mg/l (n=49)**			**Stroke with CRP ≤5 mg/l (n=124)**				
	**Median**	**Minimum**	**Maximum**	**Median**	**Minimum**	**Maximum**	**Median**	**Minimum**	**Maximum**	**Effect size η2**	**Adjusted p-value**
**HGF**	**46.0**	**3.3**	**162.0**	**94.1**	**7.1**	**2276.9**	**114.3**	**3.3**	**3244.3**	**0.085**	**0.003**
**IL-4**	**22.2**	**19.4**	**123.6**	**20.3**	**19.4**	**117.2**	**19.4**	**19.4**	**164.0**	**0.071**	**0.012**
**SDF-1α**	**1016.8**	**38.8**	**14995.3**	**1310.4**	**553.2**	**158900.0**	**1644.4**	**38.8**	**158900.0**	**0.066**	**0.022**
IL-2R	863.7	81.4	4037.1	1040.3	81.4	9822.2	1638.4	81.4	39530.9	0.046	0.175
CD30	184.1	11.6	485.2	212.0	13.2	817.1	228.7	40.6	1013.7	0.042	0.292
IL-16	104.3	13.5	703.2	177.8	13.5	1192.9	160.0	13.5	1035.9	0.041	0.319
Eotaxin-2	92.7	4.1	1070.6	138.2	6.9	2236.6	77.8	4.1	1041.8	0.029	0.999
Eotaxin	50.3	1.6	104.2	49.8	5.0	114.5	37.3	1.6	195.4	0.029	0.999
MIP-1ß	8.3	3.1	40.1	8.2	3.1	398.9	5.9	3.1	438.6	0.027	0.999
APRIL	606.4	44.1	2240.6	782.4	44.1	11668.4	772.1	44.1	35059.4	0.027	0.999
BAFF	5.7	5.7	20.8	5.7	5.7	104.5	5.7	5.7	42.2	0.023	0.999
TNF-RII	37.8	3.6	223.4	44.8	3.6	3648.5	48.4	3.6	3483.1	0.021	0.999
SCF	2.4	2.4	6.4	2.5	2.4	13.8	2.7	2.4	15.6	0.018	0.999
MIF	43.9	1.2	134.7	48.7	0.6	2450.0	52.1	1.4	2450.0	0.017	0.999
BLC	51.5	11.0	175.8	53.3	11.0	238.2	41.9	11.0	281.0	0.015	0.999
MIP-1α	2.4	2.4	49.0	2.4	2.4	52.8	2.4	2.4	98.6	0.013	0.999
Eotaxin-3	1.5	1.5	3.8	1.5	1.5	6.3	1.5	1.5	7.3	0.011	0.999
CD40L	4.7	4.7	69.9	4.7	4.7	82.0	4.7	4.7	451.8	0.010	0.999
GROα	5.6	2.4	12.6	5.3	2.4	13.7	5.0	2.4	19.0	0.008	0.999
IL-27	14.9	14.9	140.9	14.9	14.9	118.4	14.9	14.9	468.9	0.006	0.999
IP-10	25.8	2.5	86.3	29.9	5.7	140.0	28.4	2.5	129.8	0.005	0.999
MIP-3α	107.8	16.5	270.2	129.5	16.5	365.6	123.1	16.5	413.4	0.005	0.999

IL-15	3.9	3.9	21.1	3.9	3.9	9.8	3.9	3.9	86.7	0.005	0.999
IL-5	6.8	6.8	6.8	6.8	6.8	267.8	6.8	6.8	47.2	0.003	0.999
TRAIL	48.8	11.4	346.0	60.1	11.4	4520.4	47.5	11.4	6672.3	0.003	0.999
IL-18	9.3	7.0	40.8	11.8	7.0	38.5	9.8	7.0	39.8	0.002	0.999
IL-31	12.3	12.3	12.3	12.3	12.3	12.3	12.3	12.3	61.6	0.001	0.999
IL-10	2.4	2.4	2.4	2.4	2.4	6.4	2.4	2.4	14.0	0.001	0.999
MDC	97.3	18.4	241.5	116.5	18.4	520.7	97.7	18.4	293.0	0.000	0.999
LIF	4.5	4.5	17.3	4.5	4.5	24.0	4.5	4.5	40.6	0.000	0.999
IL-6	5.2	5.2	5.2	5.2	5.2	7.8	5.2	5.2	60.5	-0.001	0.999
MMP-1	610.1	15.7	19400.0	732.9	17.5	19400.0	710.4	56.0	19400.0	-0.001	0.999
M-CSF	63.4	63.4	63.4	63.4	63.4	79.2	63.4	63.4	128.5	-0.002	0.999
MCP-3	4.9	4.6	11.4	5.2	4.6	35.1	4.6	4.6	32.2	-0.002	0.999
MCP-1	38.8	3.7	191.5	46.5	3.7	185.1	47.5	3.7	310.6	-0.002	0.999
IL-2	19.3	19.3	19.3	19.3	19.3	30.4	19.3	19.3	204.4	-0.002	0.999
IL-20	7.1	7.1	31.5	7.1	7.1	58.2	7.1	7.1	125.9	-0.003	0.999
IL-8	2.0	2.0	6.6	2.0	2.0	8.7	2.0	2.0	14.7	-0.004	0.999
MCP-2	4.3	0.8	14.3	5.2	0.8	17.1	4.9	0.8	21.7	-0.004	0.999
IL-22	19.9	19.9	507.2	19.9	19.9	31924.4	20.0	19.9	81700.0	-0.004	0.999
TNF-α	4.9	4.9	4.9	4.9	4.9	14.9	4.9	4.9	6.7	-0.004	0.999
FGF-2	4.5	4.5	4.5	4.5	4.5	5.3	4.5	4.5	5.2	-0.004	0.999
I-TAC	9.7	9.7	340.2	9.7	9.7	1654.7	9.7	9.7	1571.6	-0.004	0.999
G-CSF	9.9	9.9	9.9	9.9	9.9	23.1	9.9	9.9	32.1	-0.004	0.999
GM-CSF	13.1	13.1	13.1	13.1	13.1	71.9	13.1	13.1	93.8	-0.005	0.999
MIG	8.6	8.6	232.9	8.6	8.6	5924.7	8.6	8.6	7109.7	-0.005	0.999
IL-9	10.7	10.7	10.7	10.7	10.7	11.0	10.7	10.7	58.2	-0.006	0.999
IL-1ß	3.4	3.4	5.7	3.4	3.4	4.4	3.4	3.4	8.3	-0.006	0.999
NGF-ß	4.0	4.0	4.0	4.0	4.0	4.0	4.0	4.0	5.8	-0.006	0.999

TNF-ß	6.1	6.1	6.1	6.1	6.1	6.1	6.1	6.1	20.0	-0.006	0.999
VEGF-A	208.4	17.4	688.0	219.4	5.3	2319.8	188.5	5.3	1728.8	-0.006	0.999
TSLP	6.7	6.7	40.3	6.7	6.7	7.8	6.7	6.7	65.1	-0.006	0.999
TWEAK	491.6	89.9	1550.3	435.9	89.9	1557.6	450.0	89.9	8299.1	-0.007	0.999
Fractalkine	2.6	2.6	43.3	2.6	2.6	571.2	2.6	2.6	389.7	-0.008	0.999
IL-1α	1.6	1.6	21.6	1.6	1.6	619.3	1.6	1.6	318.6	-0.008	0.999
IL-3	23.1	23.1	392.1	23.1	23.1	94700.0	23.1	23.1	94700.0	-0.009	0.999
IL-17A	23.3	23.3	83.1	23.3	23.3	50.4	23.3	23.3	478.6	-0.009	0.999
ENA-78	90.5	3.7	438.1	84.1	3.7	326.4	84.9	6.7	419.8	-0.009	0.999
IL-7	0.6	0.5	3.0	0.7	0.5	3.2	0.8	0.5	4.6	-0.009	0.999
IL-21	6.8	6.8	290.4	6.8	6.8	6212.8	6.8	6.8	12764.5	-0.009	0.999
IL-13	3.5	3.5	3.5	3.5	3.5	3.5	3.5	3.5	3.5	-0.009	0.999
IFN-α	4.8	4.8	4.8	4.8	4.8	4.8	4.8	4.8	4.8	-0.009	0.999
IFN-γ	10.7	10.7	10.7	10.7	10.7	10.7	10.7	10.7	10.7	-0.010	0.999
IL-12p70	7.6	7.6	7.6	7.6	7.6	7.6	7.6	7.6	7.6	-0.010	0.999
IL-23	16.2	16.2	16.2	16.2	16.2	16.2	16.2	16.2	16.2	-0.010	0.999

Cytokine and chemokine concentrations are shown as median, minimum and maximum concentrations in [pg/ml]. All analytes were compared between aetiology groups using Kruskal Wallis tests and p-values were corrected for 65 comparisons using Bonferroni's correction. Significantly altered analytes are highlighted in bold. Effect size coefficients η^2>0.06 indicate moderate effect sizes and η^2>0.14 indicate large effect sizes.

APRIL, a proliferation-inducing ligand; BAFF, B-cell activation factor; BLC, B-lymphocyte chemoattractant; CD30, TNF receptor superfamily member 8; CD40L, CD40-ligand; ENA78, epithelial neutrophil-activating peptide-78; FGF, fibroblast-growth factor; G-CSF, granulocyte colony-stimulating factor; GM-CSF, granulocyte-macrophage colony-stimulating factor; GRO, growth-regulated oncogene; HGF, hepatocyte growth factor; IFN, interferon; IL, interleukin; IL-2R, interleukin 2 receptor; IP, interferon-ɣ-induced protein; I-TAC, interferon-inducible T-cell α-chemoattractant; LIF, leukaemia inhibitory factor; MCP, monocyte chemoattractant protein; M-CSF, macrophage colony-stimulating factor; MDC, macrophage-derived chemokine; MIF, macrophage migration inhibitory factor; MIG, monokine induced by interferon-ɣ; MIP, macrophage inflammatory protein; MMP, matrix metalloproteinase; NGF, nerve growth factor; SCF, stem-cell factor; SDF-1α, stromal cell-derived factor-1α; TNF, tumour necrosis factor; TRAIL, TNF-related apoptosis-inducing ligand; TSLP, thymic stromal lymphopoietin; TWEAK, tumour necrosis factor-like weak inducer of apoptosis; VEGF, vascular endothelial growth factor.

Table S3. Multivariate linear regression model to predict the role of clinical and laboratory parameters on baseline plasma levels of HGF.

Variables	Estimate b (SE)	Standardised estimate ß (95% CI)	p-value
Age [years]	**0.006 (0.003)**	**0.183 (0.024 to 0.342)**	**0.024**
Sex [female-male]	-0.015 (0.067)	-0.026 (-0.257 to 0.205)	0.826
NIHSS	0.000 (0.010)	0.000 (-0.179 to 0.181)	0.993
mRS	0.011 (0.037)	0.031 (-0.170 to 0.231)	0.762
CRP >5 mg/l [yes-no]	-0.002 (0.066)	-0.004 (-0.232 to 0.225)	0.976
Ischaemic stroke [yes-no]	0.037 (0.093)	0.065 (-0.255 to 0.384)	0.690
**Aetiology**			
**LAA**	**0.609 (0.110)**	**1.068 (0.688 to 1.448)**	**<0.001**
**CE**	**0.598 (0.125)**	**1.049 (0.618 to 1.480)**	**<0.001**
**SVO**	**0.501 (0.141)**	**0.879 (0.390 to 1.367)**	**<0.001**
**sCeAD**	**Reference**		

Results are shown as estimates b with SEM (standard error of mean) and standardised estimates ß with 95% CI (confidence intervals). HGF levels were log10 transformed to meet the assumptions of the model. Samples from all 215 study participants (AIS and sCeAD-nonAIS) at baseline were included in this model. Model fit: R=0.671, R^2=0.450, F=18.3, p<0.001; collinearity: VIF<2.0, autocorrelation: R=-0.02.

AIS, acute ischaemic stroke; CE, cardioembolism; CRP, C-reactive protein; HGF, hepatocyte-growth-factor; LAA, large artery atherosclerosis; mRS, modified Rankin Scale; NIHSS, National Institutes of Health Stroke Scale; sCeAD, spontaneous cervical artery dissection; SVO, small vessel occlusion.

Table S4. Multivariate linear regression model to predict the role of clinical and laboratory parameters on baseline plasma levels of IL-4.

Variables	Estimate b (SE)	Standardised estimate ß (95% CI)	p-value
Age [years]	**0.003 (0.001)**	**0.205 (0.010 to 0.400)**	**0.039**
Sex [female-male]	-0.036 (0.029)	-0.176 (-0.459 to 0.107)	0.222
NIHSS	-0.004 (0.005)	-0.105 (-0.325 to 0.115)	0.348
mRS	0.029 (0.017)	0.221 (-0.024 to 0.466)	0.221
**CRP >5 mg/l [yes-no]**	**0.060 (0.029)**	**0.289 (0.009 to 0.569)**	**0.043**
Ischaemic stroke [yes-no]	-0.069 (0.041)	-0.333 (-0.725 to 0.058)	0.095
**Aetiology**			
**LAA**	**-0.205 (0.049)**	**-0.989 (-1.455 to -0.524)**	**<0.001**
**CE**	**-0.215 (0.055)**	**-1.041 (-1.568 to -0.513)**	**<0.001**
**SVO**	**-0.188 (0.063)**	**-0.908 (-1.506 to -0.309)**	**0.003**
**sCeAD**	**Reference**		

Results are shown as estimates b with SEM (standard error of mean) and standardised estimates ß with 95% CI (confidence intervals). IL-4 levels were log10 transformed to meet the assumptions of the model. Samples from all 215 study participants (AIS and sCeAD-nonAIS) at baseline were included in this model. Model fit: R=0.417, R^2=0.174, F=4.7, p<0.001; collinearity: VIF<2.0, autocorrelation: R=0.14. AIS, acute ischaemic stroke; CE, cardioembolism; CRP, C-reactive protein; IL-4, interleukin 4; LAA, large artery atherosclerosis; mRS, modified Rankin Scale; NIHSS, National Institutes of Health Stroke Scale; sCeAD, spontaneous cervical artery dissection; SVO, small vessel occlusion.

Table S5. Multivariate linear regression model to predict the role of clinical and laboratory parameters on baseline plasma levels of SDF-1α.

Variables	Estimate b (SE)	Standardised estimate ß (95% CI)	p-value
Age [years]	0.003 (0.003)	0.111 (-0.062 to 0.283)	0.208
Sex [female-male]	0.100 (0.062)	0.207 (-0.044 to 0.457)	0.106
NIHSS	0.011 (0.009)	0.116 (-0.079 to 0.310)	0.242
mRS	-0.034 (0.034)	-0.108 (-0.325 to 0.109)	0.328
CRP >5 mg/l [yes-no]	0.021 (0.061)	0.044 (-0.204 to 0.291)	0.727
Ischaemic stroke [yes-no]	0.050 (0.085)	0.103 (-0.244 to 0.449)	0.560
**Aetiology**			
**LAA**	**0.459 (0.101)**	**0.945 (0.533 to 1.357)**	**<0.001**
**CE**	**0.530 (0.115)**	**1.092 (0.625 to 1.559)**	**<0.001**
**SVO**	**0.571 (0.130)**	**1.176 (0.646 to 1.705)**	**<0.001**
**sCeAD**	**Reference**		

Results are shown as estimates b with SEM (standard error of mean) and standardised estimates ß with 95% CI (confidence intervals). SDF-1α levels were log10 transformed to meet the assumptions of the model. Samples from all 215 study participants (AIS and sCeAD-nonAIS) at baseline were included in this model. Model fit: R=0.595, R^2=0.354, F=12.3, p<0.001; collinearity: VIF<2.0, autocorrelation: R=0.08.

AIS, acute ischaemic stroke; CE, cardioembolism; CRP, C-reactive protein; LAA, large artery atherosclerosis; mRS, modified Rankin Scale; NIHSS, National Institutes of Health Stroke Scale; sCeAD, spontaneous cervical artery dissection; SDF-1α, stromal-cell-derived factor-1α; SVO, small vessel occlusion.

Table S6. Plasma concentrations of 65 cytokines, chemokines, and related molecules in individuals after acute ischaemic stroke classified according to the underlying stroke aetiology. Patients with cervical artery dissection leading to stroke (n=94) and those with local symptoms only (n=42) were grouped together as no differences were found between these two groups.

	Large artery atherosclerosis (n= 30)			Cardioembolism (n=34)			Small vessel disease (n=15)			Dissection (AIS and local symptoms only, n=136)			Effect size η2	Adjusted p-value
	Median	Minimum	Maximum	Median	Minimum	Maximum	Median	Minimum	Maximum	Median	Minimum	Maximum		
**HGF**	**268.3**	**42.5**	**1589.3**	**335.9**	**9.2**	**3244.3**	**250.1**	**86.2**	**788.2**	**51.7**	**3.3**	**253.0**	**0.455**	**<0.001**
**SDF-1α**	**3747.6**	**38.8**	**8782.6**	**4560.6**	**38.8**	**158900.0**	**4655.0**	**626.2**	**158900.0**	**1004.5**	**38.8**	**158900.0**	**0.469**	**<0.001**
**IL-2R**	**2953.1**	**400.3**	**39530.9**	**3001.1**	**455.6**	**9822.2**	**3732.5**	**1615.3**	**11436.6**	**681.7**	**81.4**	**4037.1**	**0.517**	**<0.001**
**CD30**	**330.9**	**151.1**	**1013.7**	**295.5**	**117.6**	**976.2**	**292.6**	**155.2**	**785.1**	**173.4**	**11.6**	**817.1**	**0.345**	**<0.001**
**TNF-RII**	**97.2**	**3.6**	**525.7**	**102.1**	**16.9**	**3648.5**	**85.9**	**47.8**	**3483.1**	**32.6**	**3.6**	**802.8**	**0.443**	**<0.001**
**IL-16**	**229.7**	**29.4**	**771.3**	**264.2**	**52.9**	**1192.9**	**270.2**	**133.6**	**960.7**	**116.6**	**13.5**	**704.9**	**0.238**	**<0.001**
**MIF**	**74.9**	**10.8**	**2450.0**	**78.3**	**15.3**	**2450.0**	**79.4**	**16.1**	**1811.2**	**43.3**	**0.6**	**154.8**	**0.194**	**<0.001**
**MIP-1ß**	**3.1**	**3.1**	**33.3**	**3.1**	**3.1**	**438.6**	**4.5**	**3.1**	**360.1**	**9.4**	**3.1**	**112.6**	**0.184**	**<0.001**
**APRIL**	**1068.9**	**123.9**	**2889.0**	**1196.0**	**276.9**	**35059.4**	**1040.9**	**364.0**	**19513.7**	**646.8**	**44.1**	**5507.0**	**0.178**	**<0.001**
**SCF**	**5.1**	**2.4**	**15.3**	**5.1**	**2.4**	**13.6**	**2.4**	**2.4**	**15.6**	**2.4**	**2.4**	**8.8**	**0.164**	**<0.001**
**IL-4**	**19.4**	**19.4**	**46.7**	**19.4**	**19.4**	**87.0**	**19.4**	**19.4**	**52.4**	**20.3**	**19.4**	**164.0**	**0.151**	**<0.001**
**GRO-α**	**2.6**	**2.4**	**9.4**	**3.6**	**2.4**	**13.5**	**3.8**	**2.4**	**9.4**	**5.8**	**2.4**	**19.0**	**0.118**	**<0.001**
**Eotaxin-3**	**1.5**	**1.5**	**6.0**	**1.8**	**1.5**	**7.3**	**1.5**	**1.5**	**3.3**	**1.5**	**1.5**	**4.5**	**0.110**	**0.001**
**IP-10**	**39.5**	**2.5**	**106.2**	**41.8**	**6.9**	**119.0**	**40.0**	**13.5**	**140.0**	**26.0**	**2.5**	**86.3**	**0.082**	**0.010**
**BAFF**	**5.7**	**5.7**	**42.2**	**5.7**	**5.7**	**9.5**	**5.7**	**5.7**	**35.2**	**5.7**	**5.7**	**104.5**	**0.072**	**0.027**
TWEAK	677.0	89.9	8299.1	627.4	133.8	7115.6	340.2	141.2	972.3	423.9	89.9	1550.3	0.065	0.051
MCP-2	6.5	0.8	21.7	5.6	1.9	14.4	6.1	2.9	17.1	4.1	0.8	14.3	0.057	0.113
MCP-1	58.5	3.7	268.5	68.5	3.7	221.1	48.3	13.3	310.6	38.5	3.7	191.5	0.036	0.963
BLC	35.0	11.0	238.2	34.9	11.0	101.9	43.8	11.0	169.4	52.5	11.0	281.0	0.033	0.999
MIP-3α	109.9	16.5	413.4	165.8	16.5	399.6	161.8	16.5	365.6	113.5	16.5	317.5	0.027	0.999
IL-27	14.9	14.9	14.9	14.9	14.9	14.9	14.9	14.9	14.9	14.9	14.9	468.9	0.027	0.999
IL-15	3.9	3.9	3.9	3.9	3.9	4.5	3.9	3.9	3.9	3.9	3.9	86.7	0.023	0.999
MMP-1	769.1	70.6	11975.3	1078.6	56.0	19400.0	1033.6	91.2	9552.8	605.7	15.7	19400.0	0.020	0.999
IL-7	0.5	0.5	2.9	0.7	0.5	3.4	0.5	0.5	3.1	0.8	0.5	4.6	0.019	0.999
G-CSF	9.9	9.9	9.9	9.9	9.9	9.9	9.9	9.9	32.1	9.9	9.9	23.1	0.014	0.999

CD40L	4.7	4.7	231.0	4.7	4.7	451.8	4.7	4.7	100.5	4.7	4.7	84.4	0.014	0.999
TNF-α	4.9	4.9	4.9	4.9	4.9	4.9	4.9	4.9	6.7	4.9	4.9	14.9	0.014	0.999
MIG	8.6	8.6	182.6	8.6	8.6	5924.7	8.6	8.6	7109.7	8.6	8.6	921.2	0.014	0.999
VEGF-A	365.2	5.3	1022.4	223.9	11.3	2319.8	260.7	13.6	922.7	176.3	8.7	1034.7	0.013	0.999
IL-18	12.0	7.0	39.4	11.5	7.0	27.6	9.2	7.0	23.8	9.4	7.0	40.8	0.013	0.999
MIP-1α	2.4	2.4	98.6	2.4	2.4	94.9	2.4	2.4	48.4	2.4	2.4	52.8	0.011	0.999
MDC	132.2	18.4	320.0	118.4	18.4	234.9	135.2	18.4	264.1	96.0	18.4	520.7	0.005	0.999
IL-8	2.0	2.0	2.0	2.0	2.0	8.7	2.0	2.0	7.4	2.0	2.0	14.7	0.002	0.999
IL-2	19.3	19.3	19.3	19.3	19.3	19.3	19.3	19.3	19.3	19.3	19.3	204.4	0.000	0.999
IL-1α	1.6	1.6	12.0	1.6	1.6	619.3	1.6	1.6	259.2	1.6	1.6	83.3	0.000	0.999
IL-20	7.1	7.1	125.9	7.1	7.1	58.2	7.1	7.1	44.1	7.1	7.1	31.5	-0.001	0.999
I-TAC	9.7	9.7	265.9	9.7	9.7	1654.7	9.7	9.7	1571.6	9.7	9.7	433.1	-0.003	0.999
FGF-2	4.5	4.5	5.2	4.5	4.5	4.5	4.5	4.5	4.5	4.5	4.5	5.3	-0.003	0.999
IL-10	2.4	2.4	2.4	2.4	2.4	2.4	2.4	2.4	2.4	2.4	2.4	14.0	-0.003	0.999
TSLP	6.7	6.7	57.2	6.7	6.7	6.7	6.7	6.7	32.1	6.7	6.7	65.1	-0.003	0.999
IL-6	5.2	5.2	5.2	5.2	5.2	17.0	5.2	5.2	5.2	5.2	5.2	60.5	-0.003	0.999
IL-5	6.8	6.8	6.8	6.8	6.8	22.0	6.8	6.8	6.8	6.8	6.8	267.8	-0.004	0.999
Fractalkine	2.6	2.6	26.4	2.6	2.6	571.2	2.6	2.6	389.7	2.6	2.6	129.0	-0.005	0.999
IL-17A	23.3	23.3	54.8	23.3	23.3	50.4	23.3	23.3	53.9	23.3	23.3	478.6	-0.005	0.999
IL-22	20.0	20.0	527.2	20.0	20.0	81700.0	20.0	20.0	10842.5	19.9	19.9	2803.8	-0.005	0.999
ENA-78	87.3	6.4	386.0	72.8	10.8	326.4	75.6	12.8	419.8	86.2	3.7	438.1	-0.006	0.999
IL-31	12.3	12.3	12.3	12.3	12.3	12.3	12.3	12.3	12.3	12.3	12.3	61.6	-0.006	0.999
IL-1ß	3.4	3.4	3.4	3.4	3.4	3.4	3.4	3.4	3.4	3.4	3.4	8.3	-0.006	0.999
Eotaxin	38.7	2.7	195.4	39.1	4.6	114.6	44.2	17.7	162.9	42.5	1.6	114.5	-0.006	0.999
IL-21	6.8	6.8	268.8	6.8	6.8	12636.4	6.8	6.8	12764.5	6.8	6.8	1876.2	-0.007	0.999
IL-3	23.1	23.1	349.7	23.1	23.1	94700.0	23.1	23.1	94700.0	23.1	23.1	3206.3	-0.008	0.999
IL-9	10.7	10.7	10.7	10.7	10.7	58.2	10.7	10.7	10.7	10.7	10.7	51.5	-0.008	0.999
GM-CSF	13.1	13.1	13.1	13.1	13.1	15.8	13.1	13.1	13.1	13.1	13.1	93.8	-0.009	0.999
MCP-3	4.6	4.6	21.6	4.6	4.6	35.1	4.6	4.6	12.3	4.9	4.6	11.4	-0.010	0.999
Eotaxin-2	78.4	4.1	2236.6	77.7	4.1	1041.8	83.4	4.1	834.1	85.9	4.1	1070.6	-0.010	0.999
LIF	4.5	4.5	33.2	4.5	4.5	10.2	4.5	4.5	11.3	4.5	4.5	40.6	-0.011	0.999
NGF-ß	4.0	4.0	4.0	4.0	4.0	4.0	4.0	4.0	4.0	4.0	4.0	5.8	-0.011	0.999
TNF-ß	6.1	6.1	6.1	6.1	6.1	6.1	6.1	6.1	6.1	6.1	6.1	20.0	-0.011	0.999

TRAIL	52.4	11.4	566.2	47.2	11.4	6672.3	57.2	11.4	5399.8	51.6	11.4	1296.6	-0.011	0.999
M-CSF	63.4	63.4	128.5	63.4	63.4	77.0	63.4	63.4	63.4	63.4	63.4	114.4	-0.012	0.999
IFN-α	4.8	4.8	4.8	4.8	4.8	4.8	4.8	4.8	4.8	4.8	4.8	4.8	-0.014	0.999
IFN-γ	10.7	10.7	10.7	10.7	10.7	10.7	10.7	10.7	10.7	10.7	10.7	10.7	-0.014	0.999
IL-12p70	7.6	7.6	7.6	7.6	7.6	7.6	7.6	7.6	7.6	7.6	7.6	7.6	-0.014	0.999
IL-23	16.2	16.2	16.2	16.2	16.2	16.2	16.2	16.2	16.2	16.2	16.2	16.2	-0.014	0.999
IL-13	3.5	3.5	3.5	3.5	3.5	3.5	3.5	3.5	3.5	3.5	3.5	3.5	-0.014	0.999

Cytokine and chemokine concentrations are shown as median, minimum and maximum concentrations in [pg/ml]. All analytes were compared between aetiology groups using Kruskal Wallis tests and p-values were corrected for 65 comparisons using Bonferroni's correction. Significantly altered analytes are highlighted in bold. Effect size coefficients η^2>0.06 indicate moderate effect sizes and η^2>0.14 indicate large effect sizes.

AIS, acute ischaemic stroke; APRIL, a proliferation-inducing ligand; BAFF, B-cell activation factor; BLC, B-lymphocyte chemoattractant; CD30, TNF receptor superfamily member 8; CD40L, CD40-ligand; ENA78, epithelial neutrophil-activating peptide-78; FGF, fibroblast-growth factor; G-CSF, granulocyte colony-stimulating factor; GM-CSF, granulocyte-macrophage colony-stimulating factor; GRO, growth-regulated oncogene; HGF, hepatocyte growth factor; IFN, interferon; IL, interleukin; IL-2R, interleukin 2 receptor; IP, interferon-γ-induced protein; I-TAC, interferon-inducible T-cell α-chemoattractant; LIF, leukaemia inhibitory factor; MCP, monocyte chemoattractant protein; M-CSF, macrophage colony-stimulating factor; MDC, macrophage-derived chemokine; MIF, macrophage migration inhibitory factor; MIG, monokine induced by interferon-γ; MIP, macrophage inflammatory protein; MMP, matrix metalloproteinase; NGF, nerve growth factor; SCF, stem-cell factor; SDF-1α, stromal cell-derived factor-1α; TNF, tumour necrosis factor; TRAIL, TNF-related apoptosis-inducing ligand; TSLP, thymic stromal lymphopoietin; TWEAK, tumour necrosis factor-like weak inducer of apoptosis; VEGF, vascular endothelial growth factor.

Table S7. Multivariate binary logistic regression model to predict the role of baseline plasma levels of altered analytes on outcome assessed a median of 2.2 (0.8-20.4) years after the acute event.

Variables at baseline	Good outcome (mRS 0-2) n=195	Unfavourable outcome (mRS 3-5) n=18	Odds ratio (95% CI)	p-value
**Age [years] [1]**	**49.4 (20.6-92.0)**	**76.5 (37.7-37.7)**	**1.08 (1.01 to 1.14)**	**0.020**
Sex (female-male)	68 (35%) – 128 (65%)	7 (39%) – 11 (61%)	1.51 (0.41 to 5.58)	0.539
**NIHSS [1]**	**1 (0-28)**	**5 (0-20)**	**1.15 (1.05 to 1.26)**	**0.002**
**CRP >5 mg/l**	**58 (30%)**	**9 (50%)**	**5.16 (1.44 to 18.51)**	**0.012**
Aetiology				
LAA	27 (14%)	3 (17%)	1.98 (0.25 to 15.77)	0.518
CE	25 (13%)	9 (50%)	1.99 (0.23 to 17.47)	0.534
SVO	12 (6%)	3 (17%)	3.12 (0.29 to 33.56)	0.348
sCeAD	132 (67%)	2 (17%)	Reference	
HGF [pg/ml] [2,3]	87.0 (3.3-3244.3)	204.5 (9.2-3203.8)	0.72 (0.25 to 2.06)	0.543 [4]
SDF-1α [pg/ml] [2,3]	1165 (39-158900)	3154 (553-76909)	1.28 (0.39 to 4.19)	0.678 [4]
IL-2R [pg/ml] [2,3]	1021 (81-39531)	2286 (249-9976)	2.39 (0.52 to 11.09)	0.266 [4]
CD30 [pg/ml] [2,3]	206.7 (11.6-976.2)	280.7 (78.1-1013.7)	7.39 (0.61 to 89.95)	0.117 [4]
TNF-RII [pg/ml] [2,3]	40.2 (3.6-3483.1)	118.7 (20.9-3649.0)	1.53 (0.47 to 4.97)	0.483 [4]
IL-16 [pg/ml] [2,3]	145.5 (13.5-997.5)	267.3 (85.8-1192.9)	1.84 (0.33 to 10.31)	0.490 [4]
MIF [pg/ml] [2,3]	46.4 (0.6-2450.0)	71.1 (10.8-2450.0)	0.76 (0.22 to 2.61)	0.662 [4]
MIP-1β [pg/ml] [2,3]	8.0 (3.1-438.6)	3.3 (3.1-398.9)	0.75 (0.21 to 2.75)	0.666 [4]
APRIL [pg/ml] [2,3]	716.3 (44-35059)	1179.0 (143-11668)	1.69 (0.43 to 6.59)	0.450 [4]
SCF [pg/ml] [2,3]	2.6 (2.4-15.6)	2.4 (2.4-13.6)	0.03 (0.00 to 0.60)	0.021 [4]
IL-4 [pg/ml] [2,3]	19.4 (19.4-164.0)	19.4 (19.4-52.4)	0.05 (0.00 to 3.64)	0.167 [4]

Results are shown as odds ratios with 95% CI (confidence intervals). [1] median (minimum-maximum); [2] data were log10 transformed to meet the assumptions of the model; [3] parameters were separately included in the model together with age, NIHSS and CRP; [4] statistically not significant ($p>0.05$) after adjustment for multiple comparisons. Model fit: McFadden's R^2=0.337, Cox&Snell's R^2=0.178, Nagelkerke's R^2=0.403, Chi-square=41.4, $p<0.001$; collinearity: VIF<2.0.

APRIL, a proliferation-inducing ligand; CD30, TNF receptor superfamily member 8; CE, cardioembolism; CRP, C-reactive protein; HGF, hepatocyte-growth factor; HGF: hepatocyte-growth-factor; IL, interleukin; IL: interleukin; IL-2R, interleukin 2 receptor; LAA, large artery atherosclerosis; MIF, macrophage migration inhibitory factor; MIP-1β, macrophage inflammatory protein 1β; mRS: modified Rankin Scale; NIHSS: National Institutes of Health Stroke Scale; sCeAD, spontaneous cervical artery dissection; SCF, stem cell factor; SDF-1α, stromal-cell-derived factor-1α; SDF-1α: stromal-cell-derived factor-1α; SVO, small vessel occlusion; TNF-RII, tumour necrosis factor receptor 2.

Table S8. Characteristics of the follow-up subgroup of ischaemic stroke patients attending both month three and one year follow-up visits, at which plasma samples were analysed for cytokine/chemokine levels.

	**Large artery atherosclerotic stroke (n=30)**	**Cardioembolic stroke (n=34)**	**Small vessel occlusive stroke (n=15)**
Female:male, n (%)	1 (3.3):29 (96.7)	10 (29.4):24 (70.6)	5 (33.3):10 (66.7)
**Month three follow-up characteristics**			
Median time from baseline until 1st follow-up [months]	3.4 (2.9-4.7)	3.2 (2.0-4.3)	3.2 (2.5-4.8)
CRP [mg/l]	0.7 (0.1-3.6)	0.2 (0.1-0.8)	0.3 (0.1-0.8)
Coagulation parameters			
INR	1.0 (0.9-1.1)	1.2 (0.9-3.8)	1.0 (0.9-1.2)
PT [%]	98.5 (78.0-117.0)	78.0 (17.0-119.0)	103.0 (74.0-114.0)
Platelet level [G/l]	246.5 (125.0-343.0)	186.0 (99.0-311.0)	223.0 (138.0-310.0)
D-Dimer [µg/l]	n.a.	n.a.	n.a.
AT-III [%]	n.a.	n.a.	n.a.
**Month twelve follow-up characteristics**			
Median time from baseline until 2nd follow-up [months]	12.6 (10.9-15.1)	12.4 (10.4-13.4)	12.6 (11.1-13.3)
CRP [mg/l]	0.1 (0.1-1.1)	0.2 (0.1-1.1)	0.2 (0.1-0.4)
Coagulation parameters			
INR	1.0 (0.9-1.1)	1.1 (0.9-2.5)	1.0 (0.9-1.2)
PT [%]	98.0 (78.0-111.0)	83.5 (39.0-120.0)	100.0 (71.0-110.0)
Platelet level [G/l]	228.5 (135.0-332.0)	188.0 (71.0-270.0)	226.5 (144.0-288.0)
D-Dimer [µg/l]	n.a.	n.a.	n.a.
AT-III [%]	n.a.	n.a.	n.a.

Values are expressed as percentages or median (min-max).
AT-III, antithrombin-III; CRP, C-reactive protein; INR, international normalised ratio; PT, prothrombin time.

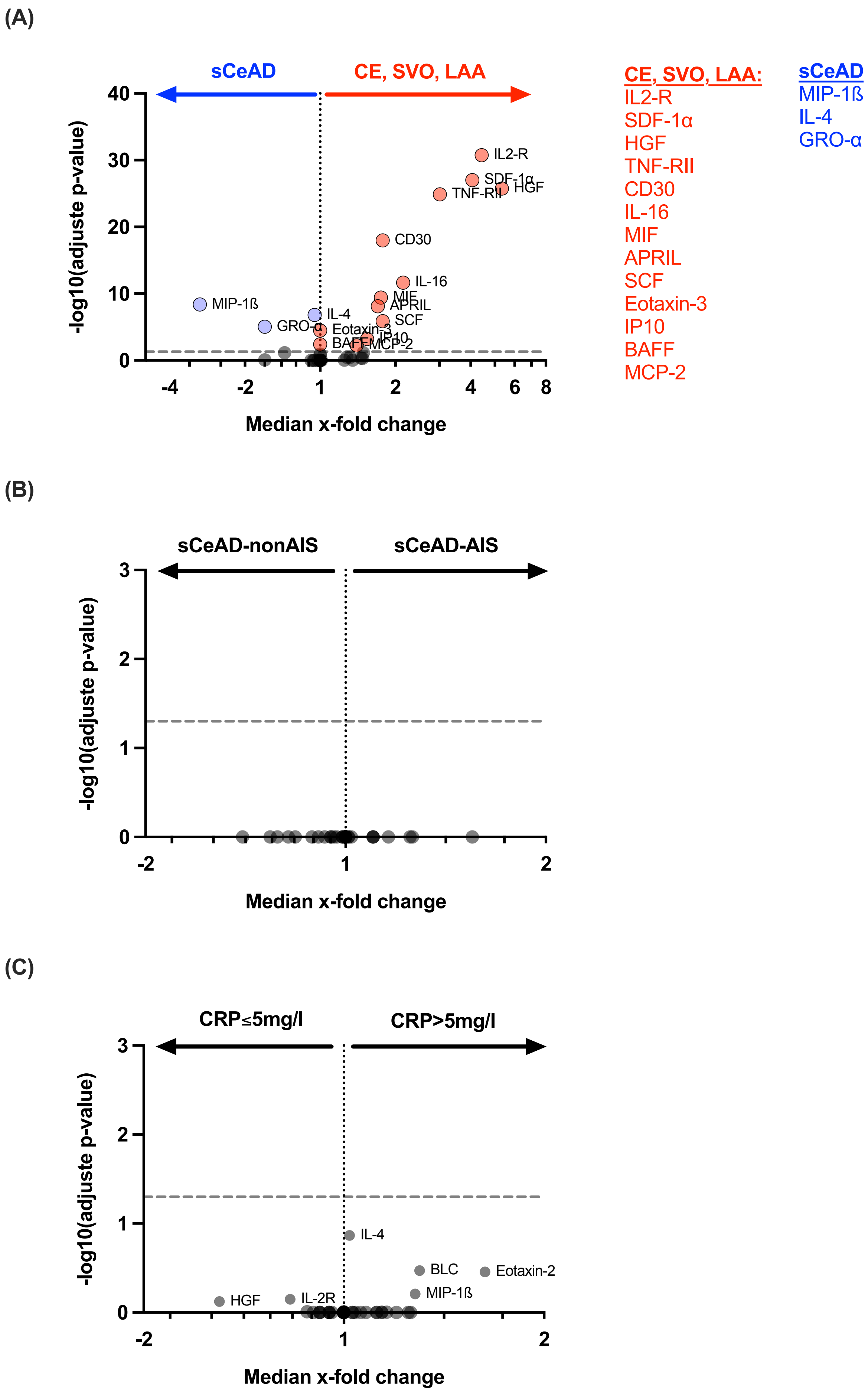
(A)
sCeAD
CE, SVO, LAA
-log10(adjuste p-value)
Median x-fold change
IL2-R
SDF-1α
HGF
TNF-RII
CD30
IL-16
MIF
APRIL
SCF
Eotaxin-3
IP10
BAFF
MCP-2
MIP-1ß
IL-4
GRO-α
CE, SVO, LAA:
IL2-R
SDF-1α
HGF
TNF-RII
CD30
IL-16
MIF
APRIL
SCF
Eotaxin-3
IP10
BAFF
MCP-2
sCeAD
MIP-1ß
IL-4
GRO-α
(B)
sCeAD-nonAIS
sCeAD-AIS
-log10(adjuste p-value)
Median x-fold change
(C)
CRP≤5mg/l
CRP>5mg/l
-log10(adjuste p-value)
Median x-fold change
IL-4
BLC
Eotaxin-2
MIP-1ß
HGF
IL-2R

Figure S1. Influence on (A) aetiology (sCeAD versus CE, SVO and LAA), (B) aetiology (sCeAD-nonAIS versus sCeAD-AIS), and (C) CRP levels >5 mg/l on plasma cytokine/chemokine concentrations. Volcano plots showing median x-fold changes versus -log10 adjusted p-values. Groups were statistically compared using multiple Mann-Whitney U tests and the significance threshold for p-values adjusted by Bonferroni's correction for 65 comparisons are indicated by the red dashed lines.

AIS, acute ischaemic stroke; APRIL, a proliferation-inducing ligand; BAFF, B-cell activation factor; CD30, TNF receptor superfamily member 8; CE, cardioembolism; GRO-α, growth-regulated oncogene-α; HGF, hepatocyte-growth factor; IL, interleukin; IL-2R, interleukin-2 receptor; IP, interferon-ɣ-induced protein; LAA, large artery atherosclerosis; MCP, monocyte chemoattractant protein; MIF, macrophage migration inhibitory factor; MIP-1β, macrophage inflammatory protein-1β; sCeAD, spontaneous cervical artery dissection; SCF, stem cell factor; SDF-1α, stromal-cell-derived factor-1α; SVO, small vessel occlusion; TNF-RII, tumour necrosis factor receptor 2.

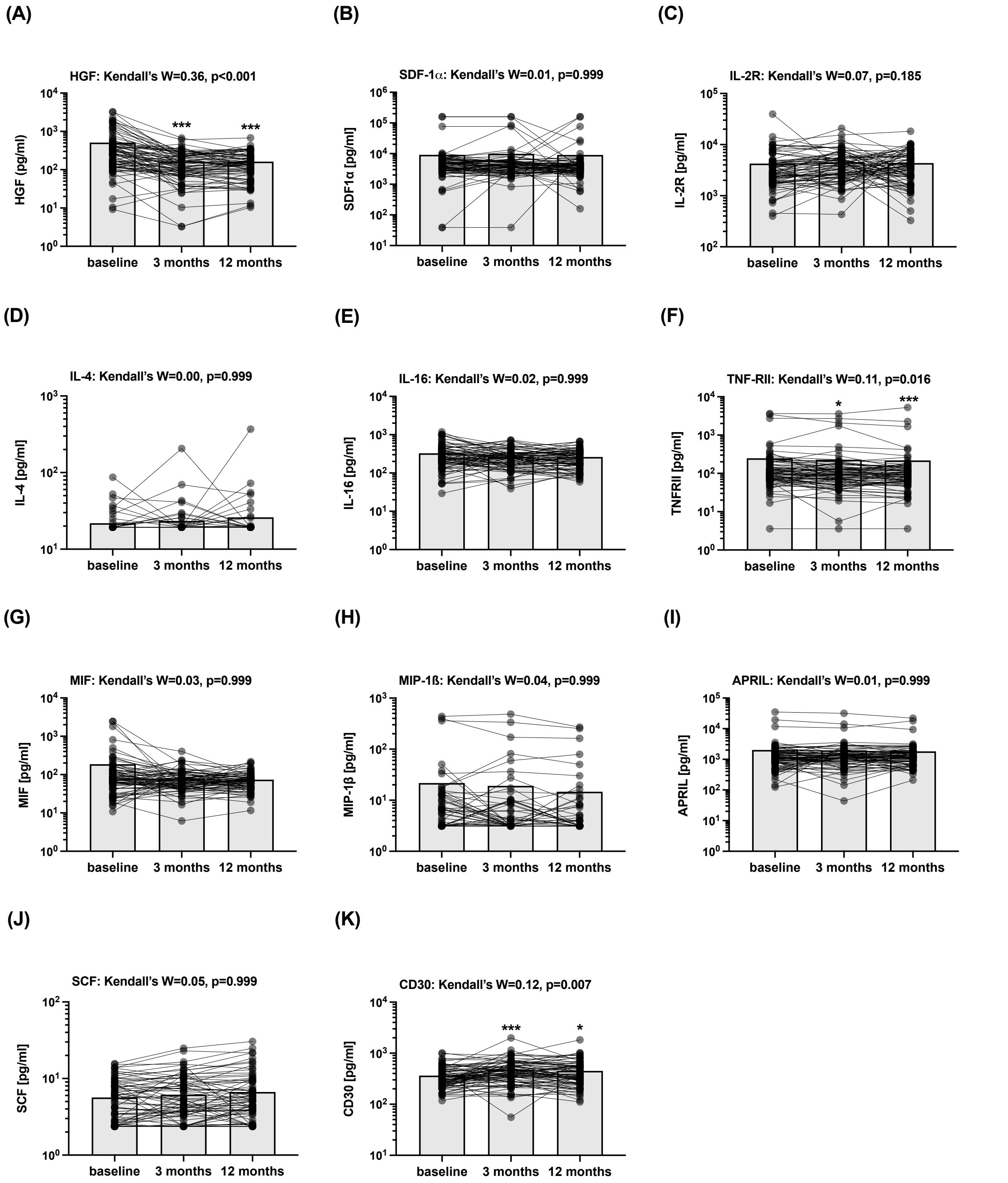

Figure S2. Plasma levels of altered cytokines/chemokines in a subgroup of people after ischaemic stroke measured at baseline, after 3 and 12 months. Plasma levels of **(A)** HGF, **(B)** SDF-1α, **(C)** IL-2R, **(D)** IL-4, **(E)** IL-16, **(F)** TNF-RII, **(G)** MIF, **(H)** MIP-1β, **(I)** APRIL, **(J)** SCF, and **(K)** CD30 were measured in a subgroup of acute ischaemic stroke patients with stroke due to large artery atherosclerosis (n=30), cardioembolism (n=34), and small vessel occlusion (n=15). Group comparisons were performed using Friedman test with Dunn's multiple comparison tests (differences to baseline: ***: p<0.001; **: p<0.01; *: p<0.05). Overall p-values were corrected for 11 comparisons using Bonferroni's correction.

APRIL, a proliferation-inducing ligand; CD30, TNF receptor superfamily member 8; HGF, hepatocyte-growth factor; IL, interleukin; IL-2R, interleukin-2 receptor; MIF, macrophage migration inhibitory factor; MIP-1β, macrophage inflammatory protein-1β; SCF, stem cell factor; SDF-1 α, stromal-cell-derived factor-1α; TNF-RII, tumour necrosis factor receptor 2.
